# Diosmin nanocrystal gel alleviates imiquimod-induced psoriasis in rats via modulating TLR7,8/NF-κB/micro RNA-31, AKT/mTOR/P70S6K milieu, and Tregs/Th17 balance

**DOI:** 10.1007/s10787-023-01198-w

**Published:** 2023-04-03

**Authors:** Yasmine Shahine, Sarah A. Abd El-Aal, Ahmed M. Reda, Eman Sheta, Nouran M. Atia, Ossama Y. Abdallah, Sherihan Salaheldin Abdelhamid Ibrahim

**Affiliations:** 1 Department of Microbiology & Immunology, Faculty of Pharmacy, Pharos University in Alexandria, Alexandria, Egypt; 2 Department of Pharmacy, Kut University College, Al Kut, Wasit 52001 Iraq; 3 Department of Biochemistry, Faculty of Pharmacy, Egyptian Russian University, Badr City, Cairo Egypt; 4 Department of Pathology, Faculty of Medicine, Alexandria University, Alexandria, Egypt; 5 Department of Pharmaceutics, Faculty of Pharmacy, Pharos University in Alexandria, Alexandria, Egypt; 6 Department of Pharmaceutics, Faculty of Pharmacy, Alexandria University, Alexandria, Egypt; 7 Department of Pharmacology and Therapeutics, Faculty of Pharmacy, Pharos University in Alexandria (PUA), Canal El- Mahmoudia Street, Smouha, Alexandria, Egypt

**Keywords:** Imiquimod-induced psoriasis, Diosmin nanocrystal gel, Th17/Treg balance, TLR7/8/NF-KB/miR-31 trajectory, AKT/mTOR/P70S6K trajectory

## Abstract

**Abstract:**

Diosmin is a flavonoid with promising anti-inflammatory and antioxidant properties. However, it has difficult physicochemical characteristics since its solubility demands a pH level of 12, which has an impact on the drug’s bioavailability. The aim of this work is the development and characterization of diosmin nanocrystals using anti-solvent precipitation technique to be used for topical treatment of psoriasis. Results revealed that diosmin nanocrystals stabilized with hydroxypropyl methylcellulose (HPMC E15) in ratio (diosmin:polymer; 1:1) reached the desired particle size (276.9 ± 16.49 nm); provided promising colloidal properties and possessed high drug release profile. Additionally, in-vivo assessment was carried out to evaluate and compare the activities of diosmin nanocrystal gel using three different doses and diosmin powder gel in alleviating imiquimod-induced psoriasis in rats and investigating their possible anti-inflammatory mechanisms. Herein, 125 mg of 5% imiquimod cream (IMQ) was applied topically for 5 consecutive days on the shaved backs of rats to induce psoriasis. Diosmin nanocrystal gel especially in the highest dose used offered the best anti-inflammatory effect. This was confirmed by causing the most statistically significant reduction in the psoriasis area severity index (PASI) score and the serum inflammatory cytokines levels. Furthermore, it was capable of maintaining the balance between T helper (Th17) and T regulatory (Treg) cells. Moreover, it tackled TLR7/8/NF-κB, miRNA-31, AKT/mTOR/P70S6K and elevated the TNFAIP3/A20 (a negative regulator of NF-κB) expression in psoriatic skin tissues. This highlights the role of diosmin nanocrystal gel in tackling imiquimod-induced psoriasis in rats, and thus it could be a novel promising therapy for psoriasis.

**Graphical abstract:**

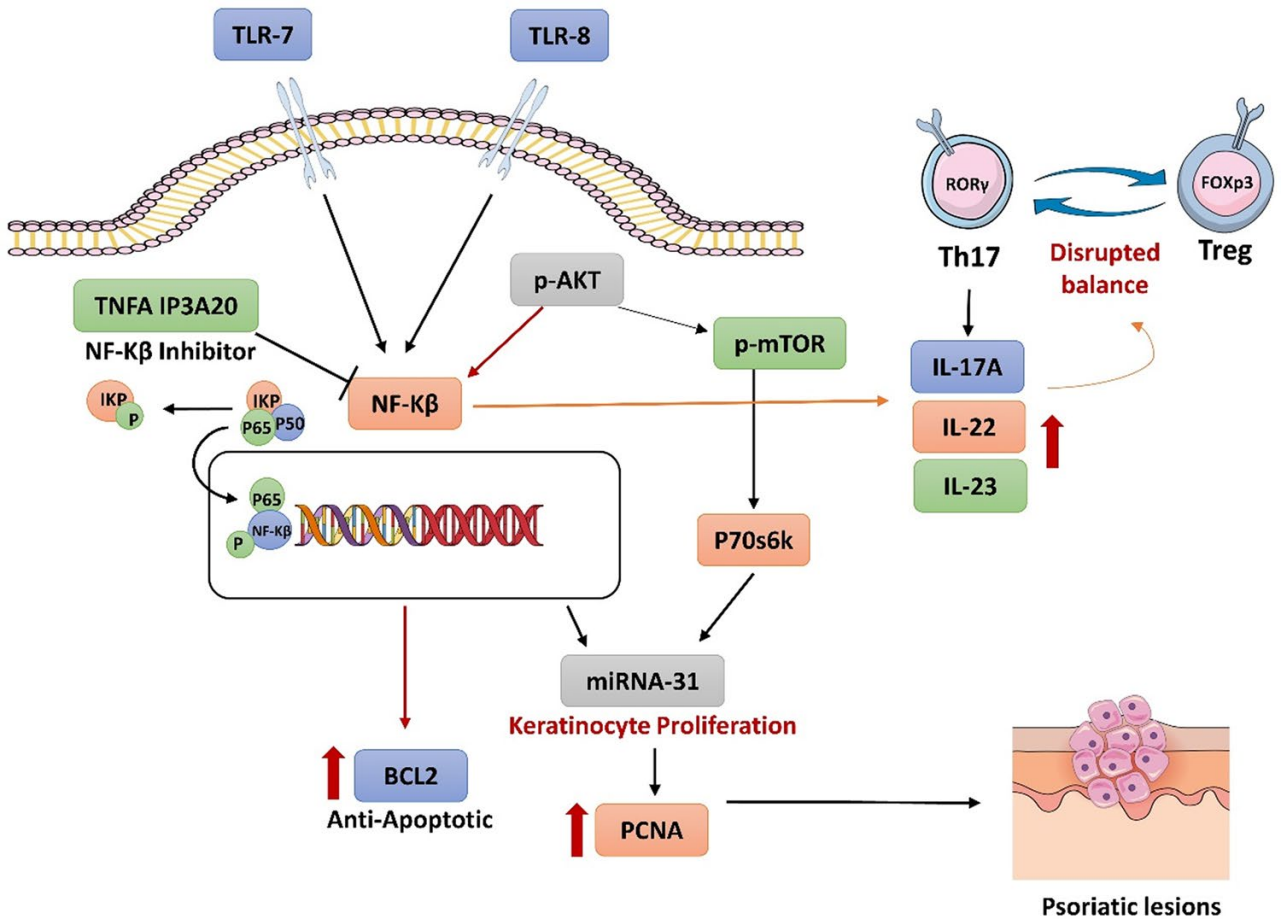

**Supplementary Information:**

The online version contains supplementary material available at 10.1007/s10787-023-01198-w.

## Introduction

Psoriasis is a chronic inflammatory skin disorder that negatively affects the patients’ quality of life. There are many factors that predispose to the occurrence of psoriasis, such as genetic factors, stress, obesity, smoking, alcohol intake, and some medicines (such as lithium, B-blockers, and anti-malarial drugs) (Branisteanu et al. 2022).

The pathophysiology of psoriasis is characterized by the interplay between many factors. The triggered immune cells in keratinocytes produce a variety of inflammatory cytokines such as tumor necrosis factor alpha (TNF-α), interleukin-1beta (IL-1β), and IL-6. These cytokines activate the plasmacytoid dendritic cells to produce interferon (IFN)-α which subsequently activates myeloid dendritic cells that produce IL-12, and IL-23. Moreover, the aforementioned cytokines, especially IL-23 activate and increase the survival of T helper 17 (Th17) to increase the production of IL-17A and IL-22 (Kjær et al. 2015). Also, it was highlighted that in psoriasis there is up-regulation of Th17 and down-regulation of regulatory T cells (Treg), which is evidenced by up-regulation of retinoic-acid-receptor-related orphan nuclear receptor gamma (ROR-ɤ) and down-regulation of fork head box P3 (FOXP-3) (Fitch et al. 2007; Shi et al. 2019).

Also, there are many dysregulated signaling pathways that are implicated in the pathophysiology of psoriasis as they can cause states of prolonged inflammation. This consequently leads to dysregulation between keratinocyte proliferation and differentiation, favoring the state of proliferation (Zhou et al. 2022). Abnormal activation of toll like receptor 7/8 (TLR7/8) is proved to be involved in the pathogenesis of psoriasis. This is proved by the ability of imiquimod (IMQ), which is a known agonist on TLR 7/8 to induce psoriasis in mice (Jiang et al. 2013). The TLR 7/8 can activate the nuclear factor kappa B (NF-κB) pathway, leading to elevation in the inflammatory cytokines and activation of IL-23/IL-17 axis (Li et al. 2013; Bender et al. 2020). Previous studies illustrated that the inhibition of activation of TLR-7/NF-κB pathway in IMQ-induced psoriasis in mice by rhododendrin played an important role in the therapy of psoriasis (Jeon et al. 2017). Additionally, it was shown that TNF-α induced protein 3, encoding A20 (TNFAIP3/A20), which is a known inhibitor of NF-κB, was mitigated in the epidermis of psoriatic patients as well as its deletion in keratinocytes caused massive elevation in the expression level of inflammatory cytokines in psoriasis (Devos et al. 2019).

Furthermore, activated NF-κB can induce microRNA-31(miR-31) that is found to be upregulated in the psoriatic patient's skin as well as in the epidermis of IMQ induced psoriasis model in mice. This induced miR-31 leads to enhanced keratinocyte proliferation in psoriasis (Yan et al. 2015). Also, it was confirmed by another study that miR-31 was augmented in keratinocytes in vivo and in vitro, subsequently it suppressed serine/threonine kinase 40 (STK40) which in turn increased the activity of NF-κB (Xu et al. 2013). This forms a positive feedback loop between miR-31 and NF-κB aggravating the pathogenesis of psoriasis.

Also it has been found that AKT signaling molecule is activated in skin of psoriatic patients and in skin of mice treated with IMQ. The activated AKT can subsequently activate mammalian target of rapamycin (mTOR) and its downstream signalling molecule p70 ribosomal protein S6 kinase (P70S6K) that can finally have a great role in elevated condition of uncontrolled epidermal cells proliferation (Mercurio et al. 2021; Karagianni et al. 2022). This is confirmed by the elevated expression of PCNA that is a marker of psoriatic epidermal proliferation (Yazici et al. 2005). Moreover, AKT indirectly induces the transcription of anti-apoptotic genes such as BCL2 via NF-κB factors (Mercurio et al. 2021; Ozes et al. 1999; Kane et al. 1999).

Traditional psoriasis treatments like phototherapy, methotrexate, cyclosporine, and acitretin, as well as medications that target T cells and cytokines, have a variety of side effects and have certain restrictions on long-term use. Furthermore, recurrence after withdrawal and decrease of efficacy are possible (Vide and Magina 2017). This necessitate the search for new therapeutic options for psoriasis.

Drug nanocrystals can be thought of as a universal formulation strategy for delivering pharmaceuticals that aren’t easily soluble. The key benefit of using nanocrystals is their ability to be administered via a variety of channels, including oral, parenteral, and ocular methods. Unfortunately, there are few researches done addressing their topical route of administration (Gigliobianco et al. 2018; Lv et al. 2022).

A flavonoid known as diosmin exhibits potent anti-inflammatory and antioxidant effects. Due of its extremely unfavourable physicochemical characteristics concerning solubility and bioavailability, only a small number of documented research used this intriguing medication (Huwait and Mobashir 2022; Gerges et al. 2022).

Therefore, the aim of this work is to enhance the dissolution behaviour of diosmin via development of nanocrystals using anti-solvent precipitation technique as well as evaluate different stabilizers with different concentrations to achieve the most stable nanocrystals. Then use the prepared diosmin nanocrystals gel topically in 3 different concentrations to evaluate its efficacy in alleviating IMQ induced psoriasis in rats via modulating TLR7,8/NF-κB/micro RNA-31, AKT/mTOR/P70S6K milieu and Tregs/Th17 balance. Finally, contrast the effectiveness of the diosmin nanocrystal gel with the diosmin powder gel used in the same experimental model.

## Materials and methods

### Part 1

#### Preparation of diosmin nanocrystals

##### Materials

Diosmin (Jianshi Yuantong Bioengineering Co., Ltd, China), hydroxypropyl methylcellulose (HPMC E15) and methyl cellulose (MC) (Zhengzhou Tianying Chemicals Company, China), Poloxamer 407 (Kolliphore 407) (BASF chemical company, Ludwigshafen, Germany), sodium alginate (Qingdao Lanneret Biochemical Co., Ltd., China), dimethyl sulphoxide (DMSO) (Oxford chemicals, India), potassium dihydrogen ortho phosphate, sodium hydroxide and Boric acid (El-Nasr pharmaceutical Company, Egypt), dialysis tubes, Visking^®^ 36/32, 28 mm, MWCO 12,000–14,000 (Serva, USA).

##### Method of preparation of diosmin nanocrystals

Diosmin nanocrystals were prepared using anti-solvent precipitation technique. Diosmin (150 mg) was dissolved in (5 ml) dimethyl sulphoxide (DMSO) (solvent phase). The organic solution was added into aqueous solution of distilled water (anti-solvent phase) containing different stabilizers in different concentration under continuous stirring. Stirring was maintained at 500 rpm for 30 min after addition till a homogenous hazy dispersion was obtained. The Stabilizers used were; HPMC E15, methyl cellulose (MC) and Poloxamer 407. Each of them was used alone or in a mixture.

#### Characterization of diosmin nanocrystal formulations

##### Particle size analysis using Malvern Zetasizer

Dynamic light scattering using Malvern Zetasizer was used to investigate the average particle size of the prepared diosmin nanocrystal formulations. Samples were diluted with distilled water previously filtered using 0.22 μm millipore filters and sonicated for 5 min. All measurements were performed in triplicates.

##### Optical light microscopy

All the prepared formulations (F1–F11) were observed and assessed using optical light microscopy. Examination using optical light microscope could be considered a short term stability study in order to show the ability of the nanocrystals to maintain their shape and size with time. All images were observed immediately after preparation and after storage at room temperature (25 °C) and refrigerator (4 °C) for seven days under magnification 400X (Supplementary Table 1).

##### In-vitro drug release study

In-vitro release of diosmin from different nanocrystal formulations using dialysis bag technique (Sink condition): In-vitro drug release was tested for selected diosmin nanocrystal formulations (F2, F6, F7, F8, F11) and control (non-nanonized diosmin). Dialysis of an amount equivalent to 2.7 mg diosmin was carried out through cellophane bag 5 cm long tied from both edges, with a 28 kDa cut off (VISKING dialysis tubing, SERVA, electrophoresis, Germany). The bags were transferred to ambered glass bottles containing 10 ml of orthophosphate buffer pH 12, in horizontal shaking water bath at 100 rpm, and temperature was adjusted to 37 ± 0.5 °C. The total volume was withdrawn at different time intervals (0.25, 0.5, 0.75, 1, 2, 3, 4, 5 and 6 h) and replaced with an equivalent volume of a previously warmed fresh medium. Samples were diluted with the dissolution medium and measured spectrophotometrically at *λ* max 266 nm. The percentage release was calculated on the cumulative drug released at different time intervals. All measurements were performed in triplicates.

In-vitro release of diosmin from different nanocrystal formulations using dissolution USP type II apparatus: (Non-Sink condition): Dissolution experiment was conducted for the previously selected formulations as in pH 12. The experiment was performed in a dissolution USP type II apparatus (paddle method). The dissolution medium consisted of 500 ml borate buffer pH 10. The amount of sample for each experiment was equivalent to 3 mg diosmin. Dissolution experiments were performed at 37 ± 0.5 oC with a paddle speed 100 rpm. Samples of 5 ml were withdrawn at predetermined time intervals (5, 10, 15, 30, 45, 60, 90, 120 and 180 min), and then replaced by an equal volume of fresh dissolution media. The solution was then filtered through 0.22 μm millipore filter and analyzed spectrophotometry at *λ* max 266 nm (Atia et al. 2019).

##### Transmission electron microscopy

Selected diosmin nanocrystal formulation (F2) was examined using a transmission electron microscope (TEM). Prior to TEM examination the nanocrystals was diluted with distilled water previously filtered and sonicated for 10 min. One drop of the diluted dispersion was placed on a copper coated grid leaving a thin film, followed by air drying. The dried film was then viewed on a TEM and photographed.

##### Fourier-transformed Infrared spectroscopy

The fourier transform—infrared (FT-IR) spectra for diosmin, HPMC E15, F2 and physical mixture for this formulation were recorded using an FT-IR spectrometer. A sufficient amount (approximately 2–4 mg) of the sample was placed to form a thin film covering the diamond window. The FT-IR spectra were recorded at spectral resolution of 2 cm ^−1^ with an average of 20 scans.

##### X-ray diffraction (XRD)

The crystallinity of diosmin in the selected nanocrystal formulation was assessed by X-ray powder diffraction (XRD). A copper radiation source was used as the anode material. The diffraction pattern was performed in a step scan model with a voltage of 40 kV and a current of 40 mA in the range of 10° < 2Ɵ < 40°. Samples investigated were as follows; crude diosmin and F2.

#### Preparation of diosmin nanocrystal gel

The selected diosmin nanocrystal formulation (F2) which was stabilized with HPMC E15 in concentration (Diosmin:Stabilizer 1:1) as previously described, was thickened using sodium alginate in concentration 2% (weight ratio) to prepare a gel formulation.

#### Characterization of diosmin nanocrystal gel

##### Particle size analysis using Malvern Zetasizer

In order to investigate the effect of gel formation on the average particle size measurements. The average particle size of the formulated gel was measured using Malvern Zetasizer.

##### In-vitro release testing at 32 °C

The formulated nanocrystal in a gel form was selected for the in-vivo study via topical route of administration. Un-expected release behaviour might occur during diosmin release at skin temperature (32 °C). Therefore, further confirmation for the release study was done at 32 °C for the selected formulation in a form of a gel and diosmin powder gel.

### Part 2

#### Animals

Forty-eight adult female Sprague–Dawley rats were 12–14-week old weighing (150–200 gm). They were obtained from the animal house of Pharos University in Alexandria. Before the experiment, rats were left for one week with free access to pelleted standard rat chow and water to accommodate with the surrounding environment. All experimental procedures were performed according to the guide for the Care and Use of Laboratory Animals (NIH), the Animal Research: Reporting of In Vivo Experiments (ARRIVE) guidelines, and approved by the “Unit of research Ethics Approval committee, Pharos University in Alexandria” (PUA-01202002233004).

#### Induction and experimental groups

**Fig. 1:**
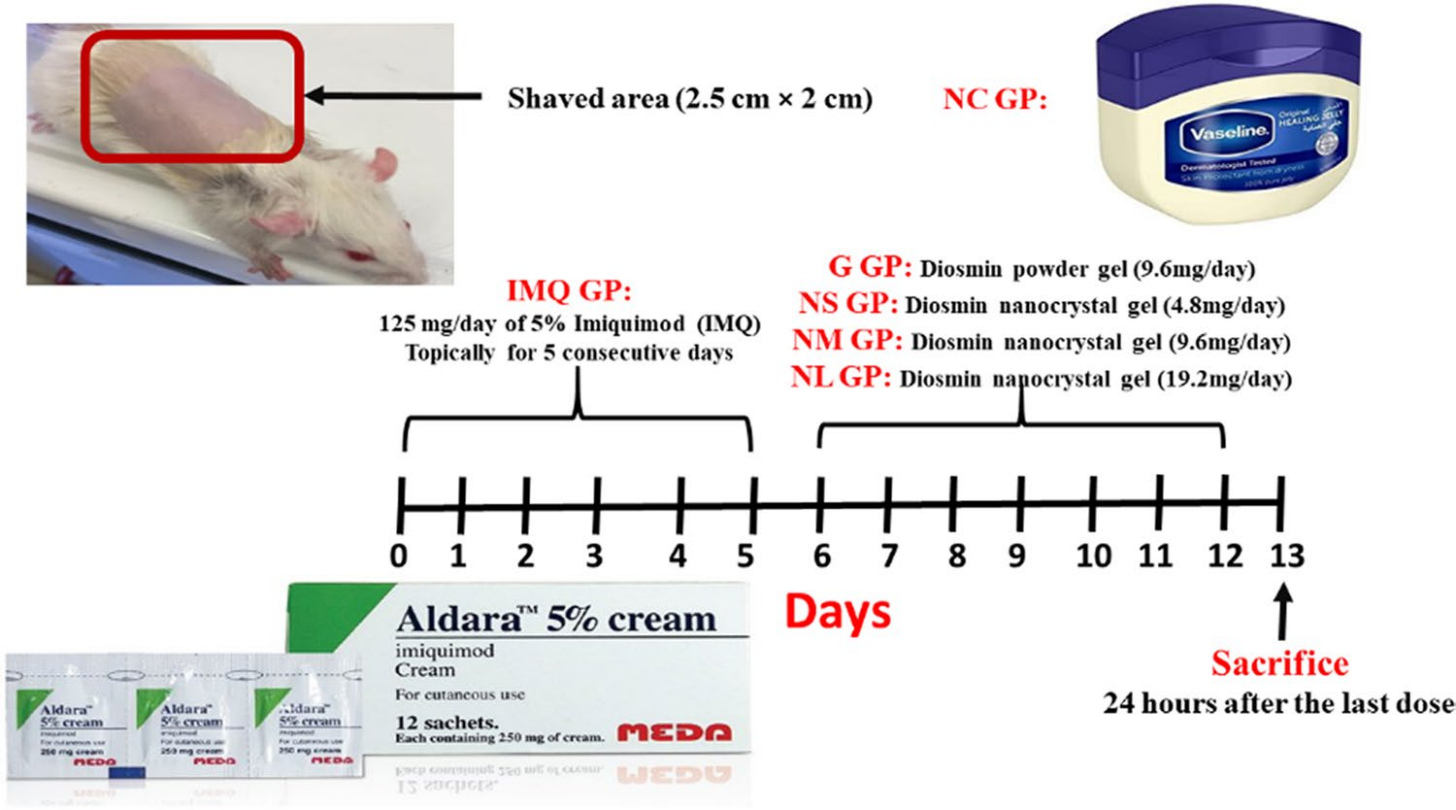
Experimental design of the imiquimod (IMQ)-induced psoriasis in rats

The experimental design of imiquimod-induced psoriasis in rats is illustrated in Fig. 1. At the beginning of the study animal backs were shaved (2.5 cm × 2 cm) (Sakai et al. 2016). Animals were allocated randomly into 6 different groups (*n* = 8). Group 1 Negative control group, rats were treated with a control vehicle cream (Vaseline cream, Unilever). In the 5 remaining groups induction of psoriatic like rat model was done by applying 125 mg of 5% imiquimod cream (Aldara; Meda Pharmaceuticals, Switzerland), containing 6.25 mg of IMQ, to the shaved skin on the back of rats and continued for 5 consecutive days (Smajlović et al. 2021). Group 2 IMQ-control group (positive control) is treated with placebo gel. Group 3 was treated with 2 ml of diosmin powder gel on their back at a dose of 9.6 mg/day/rat. Three groups from 4 till 6 received the allocated treatment with 2 ml of diosmin nanocrystal gel formulation on their back at doses of 4.8, 9.6 and 19.2 mg/day/rat, respectively. All rats were given the treatments were applied topically for 7 consecutive days. At the end of the experiment, photos were taken to identify the changes in the back skin.

#### Psoriasis area severity index (PASI) assessment

The Psoriasis area severity index (PASI) was assessed to indicate the psoriatic lesion severity (Okasha et al. 2018). Scores ranging from 0 till 4 were given for erythema, thickening and scaling where (0 indicates none, 1 indicates slight, 2 indicates moderate, 3 indicates marked and 4 indicates very marked). The total score for each group was calculated that ranges from 0 to 12. By the end of the study, rats were anesthetized by ketamine and xylene. Then the rats were sacrificed and blood was collected from aorta after 24 h from the final treatment. Serum was separated and stored at − 80 °C until analysis. Also, central dorsal skins of the rats were isolated and cut into 2 halves, one half is kept at − 80 °C for analysis of tissue parameters and the other half is kept in formalin for further embedding in paraffin blocks followed by histopathological and immunohistochemical investigations.

#### Serum parameters

Assessment of serum IL-17A (cat no# ab214028, Abcam, USA), IL-23 (cat no# ERA28RB, Invitrogen, Thermofisher scientific, USA), and IL-22 (cat no# ERA27RB, Invitrogen, Thermofisher scientific, USA) concentrations were measured by using ELISA kits according to the manufacturer instructions.

#### Western blot analysis

The central dorsal skins were homogenized with RIPA lyzing buffer. Then this was followed by centrifugation at 4 °C for 10 min at 12,000 rpm to obtain total protein. The supernatant was obtained and used for colorimetric quantitation of total protein by using bicinchoninic acid assay (Thermo Fisher Scientific). Then protein samples were run in 10% SDS-PAGE gel and electro-transferred to a polyvinylidene difluoride membrane. The membrane was blocked with TBST containing 5% skim milk for 1 h at room temperature and subsequently incubated with the primary antibodies at 4 °C overnight, Table 1. Then the membrane was incubated with a secondary antibody, HPR-conjugated goat anti-rabbit IgG for 1 h at room temperature at room temperature. Finally, blots were detected by a Bio-Rad Gel imaging system and analyzed by Image J software.

**Table 1:** Summary for the used primary antibodies in western blot technique:

Primary antibody against	Kit used
PCNA	Cat no# ab152112, Abcam, USA
BCL2	Cat no# ab196495, Abcam, USA
AKT (phospho T308)	Cat no# ab38449, Abcam, USA
mTOR (phospho Ser2448)	Cat no# 2971, Cell signaling technology
P70S6k (phospho Thr389)	Cat no# 9205, Cell signaling technology
TNFAIP3(A20)	Cat no# PA5-20729, Invitrogen, Thermofisher scientific, USA
TLR 7	Cat no# PA1-28109, Invitrogen, Thermofisher scientific, USA
TLR 8	Cat no# ab180610, Abcam, USA
NF-kβ-p65 (phospho Ser468)	Cat no# PA5-35597, Invitrogen, Thermofisher scientific, USA

#### RNA isolation and quantitative reverse transcription-polymerase chain reaction (qRT-PCR)

Central dorsal skin was harvested from rats in different groups. Total RNA was extracted from excised skin tissues using TRIzol Plus RNA Purification Kit (Cat no# 12183555, Invitrogen, USA), according to the manufacturer’s protocol. Then, the RNA samples were converted to the cDNA by reverse transcription at 37 °C for 60 min subsequently amplified, using the Superscript One-Step RT-PCR System (Cat no.12594100, Invitrogen, USA) with gene-specific oligonucleotide primers, Table 2. Amplification curves and Ct values were determined by Stratagene MX3005P software. To estimate the variation of gene expression on the RNA of the different samples, the Ct of each sample was compared with that of the positive control group. Relative miR-31 expression level was calculated using the “2-ΔΔCt” method, first: Δ Ct = Ct assessed gene − Ct reference gene, then: Δ Δ Ct = Δ Ct sample − Ct internal control gene, and finally: RQ = 2 − (Δ Δ Ct).

**Table 2:** All primer pairs used possessed 97% efficiency and primers sequences used were as follows:

Gene	Forward sequence	Reverse sequence
miR-31	5′-ACGCGGCAAGATGCTGGCA-3′	5′-CAGTGCTGGGTCCGAGTGA-3′
β-actin	5′-ATGATATCGCC GCGCTCG-3′	5′-CGCTCGGTGA GGATCTTCA-3′

#### Histopathological and immunohistochemical analysis

Assessment of H&E stained sections from lesional skin of different studied groups was done under light microscopy (Olympus, CXR-23). Full epidermal thickness was measured in microns by recording the distance between the top of the epidermis and the bottom of the rete ridges. At least four different measurements were recorded in each sample and then average was calculated.

Immunostaining by both FOXP3 monoclonal antibody (Thermo Fisher Scientific, Cat # 14-5773-82) with a dilution of 1:200 and RORγ polyclonal antibody (Bioss, Cat # bs-6217R) with a dilution of 1:200 was done using Envision detection system (Dako autostainer Link48) applying DAB as chromogen and hematoxylin as a counterstain. Positive lymphocytes within dermal inflammation were counted per one high power field (HPF) using Image J software. Then the mean number/ 5 HPFs was calculated (Fondi et al. 2009).

#### Statistical analysis

Statistical analysis of data (in the in vivo study) was carried out using one-way analysis of variance ANOVA followed by Tukey’s post hoc test for parametric data, while Benferroni test was used for nonparametric data. The software employed was GraphPad Prism Version 8. Values are expressed as mean ± standard deviation (mean of 8 values/group). Using the Spearman coefficient test, statistical correlations between different parameters were performed by IBM SPSS software package version 20.0 (Armonk, NY: IBM Corp). The level of significance was set at *P* ≤ 0.05.

## Results

### Part 1

#### Particle size analysis and morphological examination

Table 3 shows the different particle size in polydispersity index (PDI) of different formulations prepared with different stabilizers. In addition, diosmin needle-shaped crystals were observed over time indicating crystal growth of nanoparticles. Growth of diosmin crystals was more obvious when nanocrystals were kept at room temperature compared to those stored in refrigerator, (Supplementary Table 1).

**Table 3:** Particle size (PS) and polydispersity index (PDI) of diosmin nanocrystal formulations measured by Malvern Zetasizer at 25 °C

Formulation Code	Composition	Diosmin: Stabilizer Ratio	PS (nm) ± SD	PDI ± SD
F 1	HPMC E15	1:0.5	350.40 ± 27.76	0.62 ± 0.03
F 2	HPMC E15	1:1	276.90 ± 16.49	0.43 ± 0.02
F 3	HPMC E15	1:2	257.00 ± 17.09	0.62 ± 0.03
F 4	Poloxamer 407	1:0.5	473.40 ± 15.76	0.70 ± 0.02
F 5	Poloxamer 407	1:1	436.90 ± 15.04	0.73 ± 0.02
F 6	Poloxamer 407	1:2	567.70 ± 19.18	0.68 ± 0.04
F 7	MC	1:0.5	347.40 ± 12.36	0.47 ± 0.03
F 8	MC	1:1	295.80 ± 9.77	0.44 ± 0.04
F 9	HPMC E 15: Poloxamer 407	1:(0.5:0.5)	266.60 ± 10.70	0.59 ± 0.03
F 10	HPMC E 15: Poloxamer 407	1:(1:1)	301.10 ± 14.97	0.64 ± 0.02
F 11	MC:Poloxamer 407	(1:1):1	474.50 ± 24.70	0.53 ± 0.03

Based on the average particle size and optical light microscope results F2, F6, F7, F8 and F11were selected for further in- vitro release study, as these formulations showed accepted average particle size and the most delaying in appearance of aggregates and needle-shaped crystals.

#### In-vitro drug release study in sink condition pH 12 using dialysis bag method

**Fig. 2:**
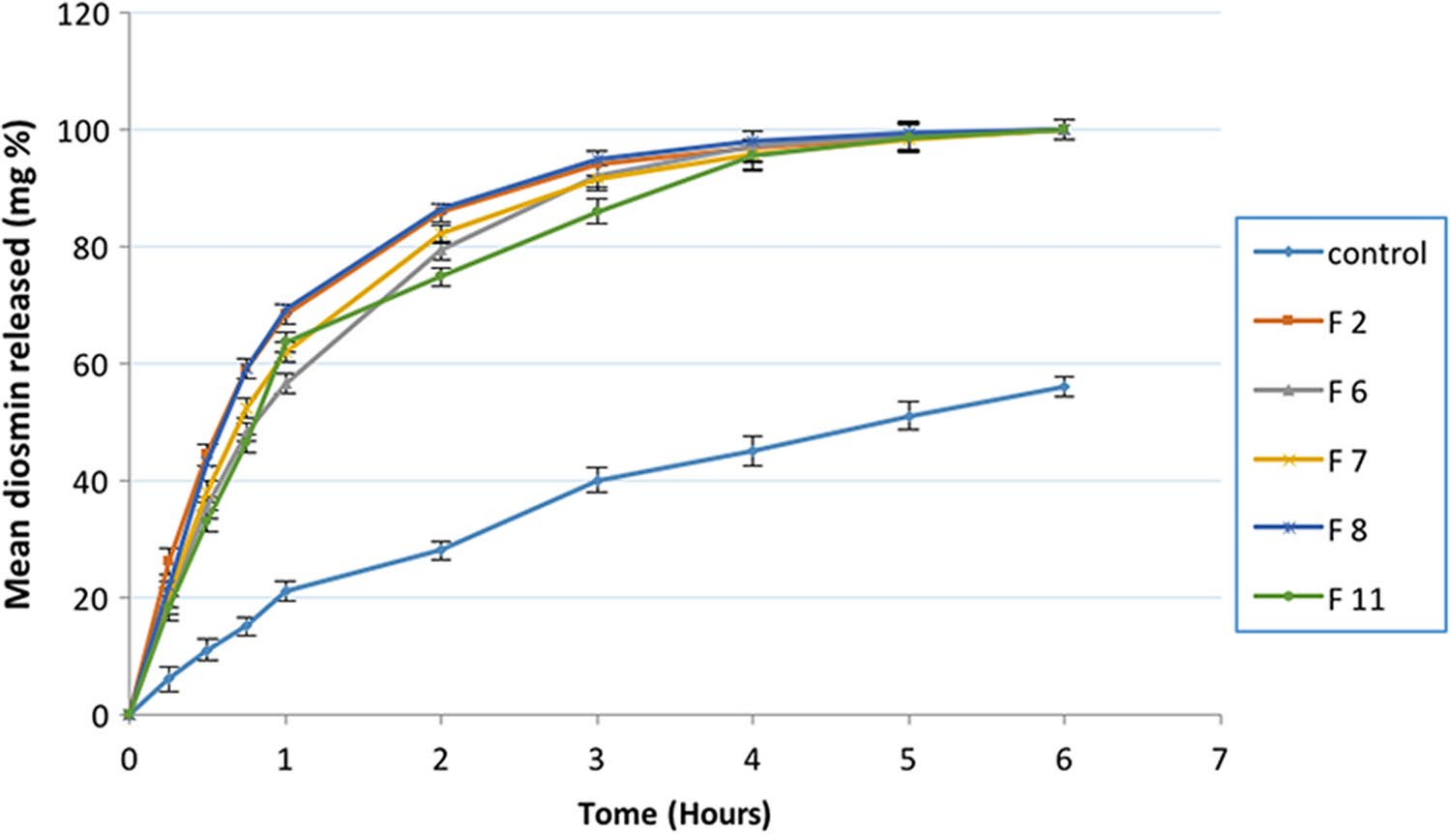
In-vitro release profile of different diosmin nanocrystals by dialysis bag method (dissolution medium: 10 ml orthophosphate buffer pH 12, 100 rpm, 37 °C). Data presented as mean (of triplicates) ± SD

The release study was conducted in pH 12 through dialysis bag method and the results are shown in Fig. 2. The percent diosmin diffused from all nanocrystal formulations were approximately more than 50% in the initial 45 min compared to about 10% for the non-nanosized drug (control). Thus, there was a significant increase in % diosmin released in case of nanocrystal formulations (*P* = 0.0017, < 0.05). As shown in Fig. 2, the use of sink condition in release testing for nanocrystal led to rapid release rates. Thus, it caused difficulties in discrimination between different formulations so nearly the same release profiles were observed for different formulations. Therefore, non-sink conditions were recommended for the dissolution testing of poorly water-soluble drug nanocrystals for better discrimination between different formulations.

#### In-vitro drug release study in non-sink condition pH 10

**Fig. 3:**
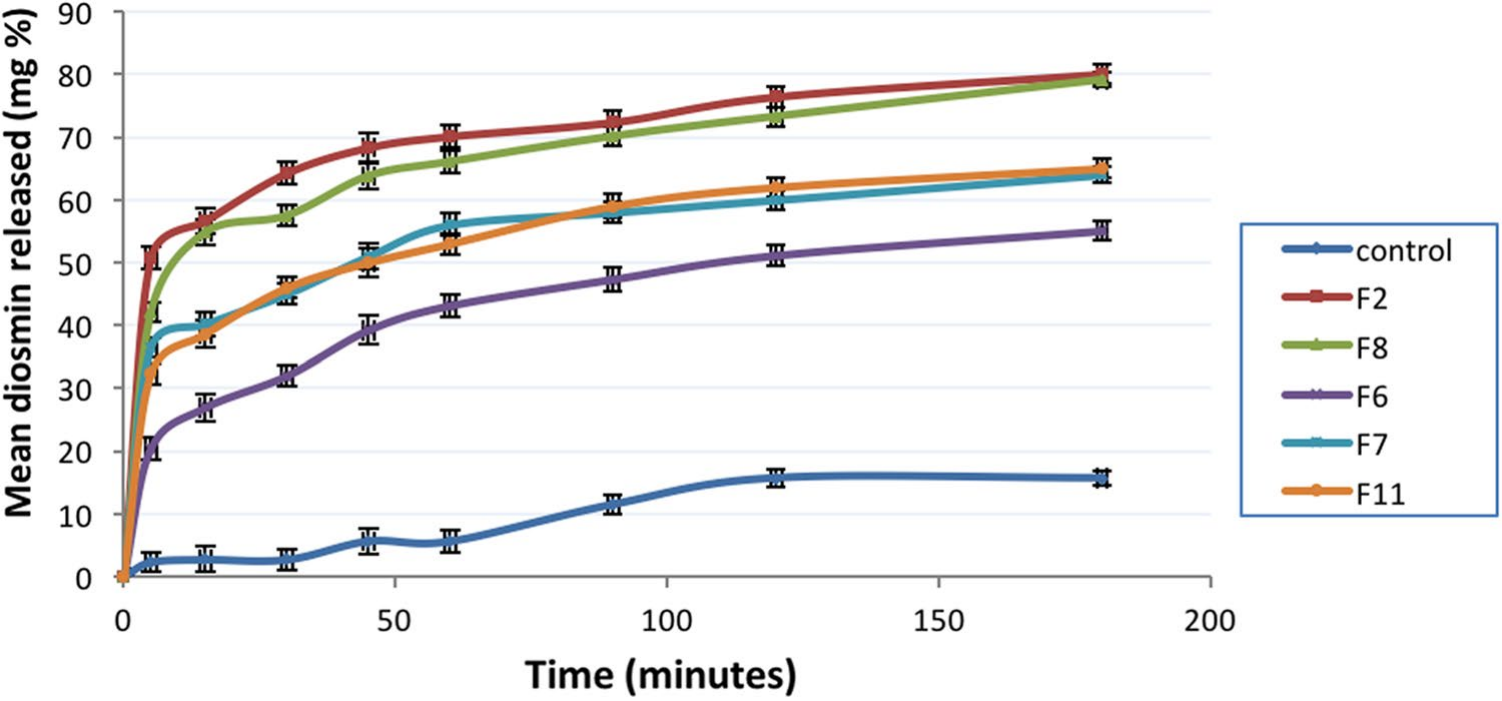
In-vitro release profile of different diosmin nanocrystals by dissolution method (dissolution medium: 500 ml borate buffer pH 10, 100 rpm, 37 °C). Data presented as mean (of triplicates) ± SD

As shown in Fig. 3, selected diosmin nanocrystal formulations were compared with the control nanocrystals. A significant difference in dissolution rate between nanocrystal formulations and the control formulation were observed (*P* = 0.00164, < 0.05). The dissolution rate in control nanocrystal did not exceed more than 15% over 3 h. There was an initial burst effect that occurred within the first thirty minutes and the remaining amount was released in a sustained release manner.

Furthermore, the effect of different stabilizers in different concentrations on the release profile of diosmin nanocrystal was assessed by comparing the dissolution profile for five different formulations., F2 stabilized with HPMC E15 in concentration (diosmin:stabilizer 1:1), showed higher dissolution rate where 50% of drug was dissolved in the first five minutes. These results are in correlation with the particle size analysis and optical microscope images. F2 showed no needle-shaped crystals or aggregates when monitored using optical light microscope with average particle size 276.9 nm. Based on the previous results, F2 was the most stable formulation with higher dissolution rate, making it a suitable candidate for further experiments and for in vivo study.

#### Transmission electron microscopy

**Fig. 4:**
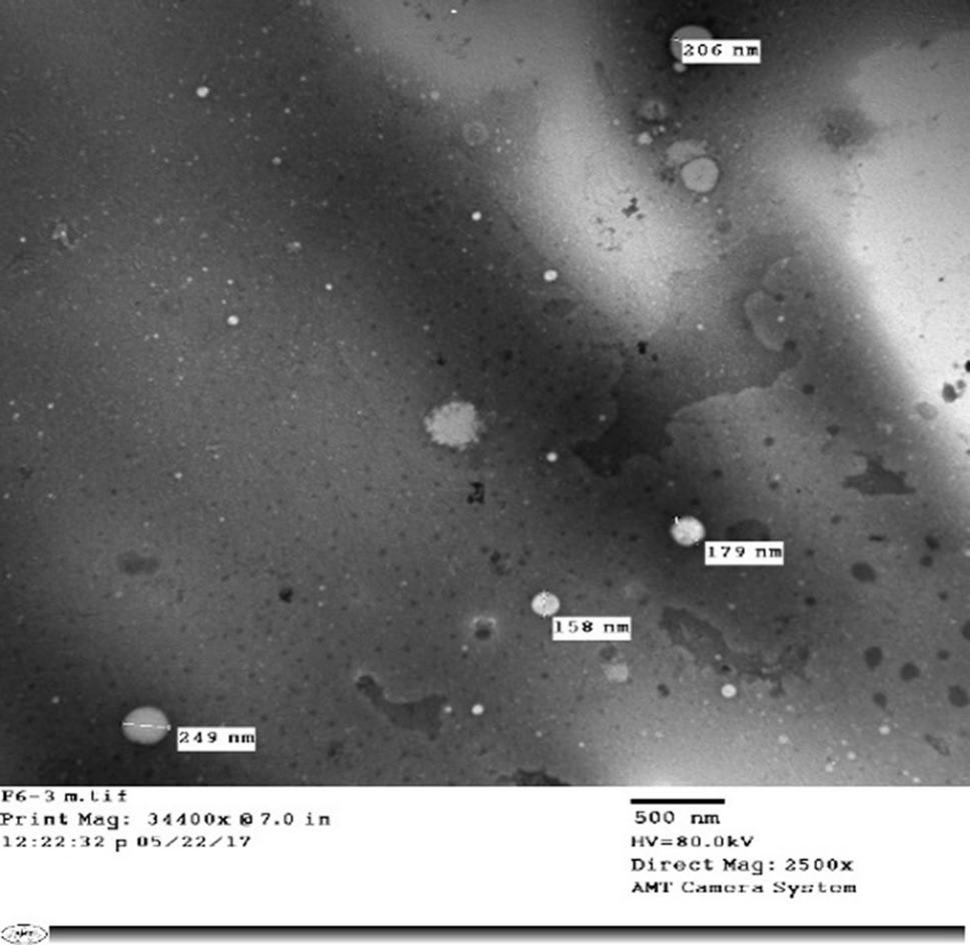
TEM micrograph of diosmin nanocrystal formulation (F2)

The morphology of diosmin nanocrystal formulation F2 was investigated using TEM, Figure 4. Spherical diosmin nanoparticles (158–249 nm) were formed. Particle size results were lower than the mean data obtained from particle size analysis.

#### Fourier-transformed infrared spectroscopy (FT-IR)

**Fig. 5:**
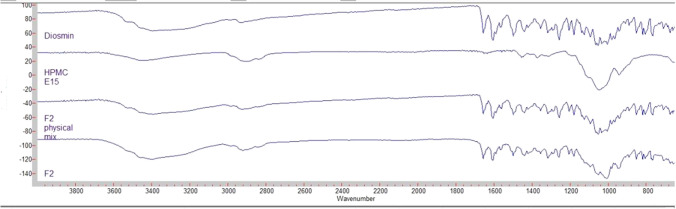
IR spectrum of diosmin, HPMC E15, F2 and F2 physical mixture

Fourier transform infrared spectroscopy has been used to detect any interaction between the drug and stabilizer. The FT-IR spectra of diosmin powder, HPMC E15, diosmin/HPMC (1:1) physical mix and the selected formulation (F2) were shown in Figure. The IR spectrum of F2 didnʼt exhibit any changes in the structure in comparison with their corresponding physical mixtures. Therefore, there was no interaction between drug and stabilizer upon nanoperciptation process, Fig. 5.

#### X-ray diffraction analysis

**Fig. 6:**
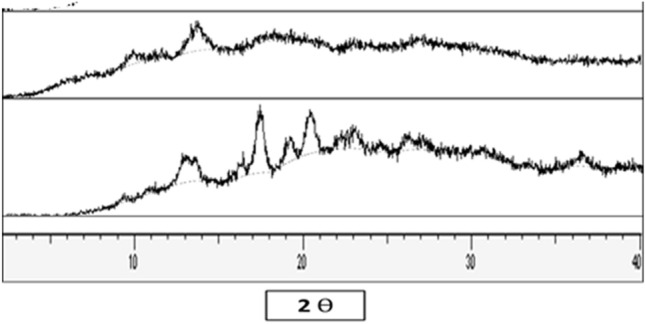
X-ray diffraction patterns of coarse diosmin powder (bottom) and F2 (top)

The x-ray diffraction patterns of coarse diosmin and F2 are shown in Fig. 6. The diffraction pattern of diosmin powder revealed high sharp-intensity peaks, suggesting the crystalline nature of the drug. The diffraction peaks of F2 showed that the formulation was partially amorphous as there was a decrease in the intensity of diosmin characteristic peaks.

#### Characterization of diosmin nanocrystal gel

##### Particle size analysis using Malvern Zetasizer

In order to investigate the effect of gel formation on the average particle size measurements. The average particle size of the formulated gel was measured using Malvern Zetasizer. Incorporation of diosmin nanocrystals stabilized by HPMC E15 in gel matrix resulted in average particle size 320.5 nm. When these result was compared with F2 nanocrystals (276.9 nm), it showed that there was no much increase in particle size.

##### In-vitro release testing at 32 °C

**Fig. 7:**
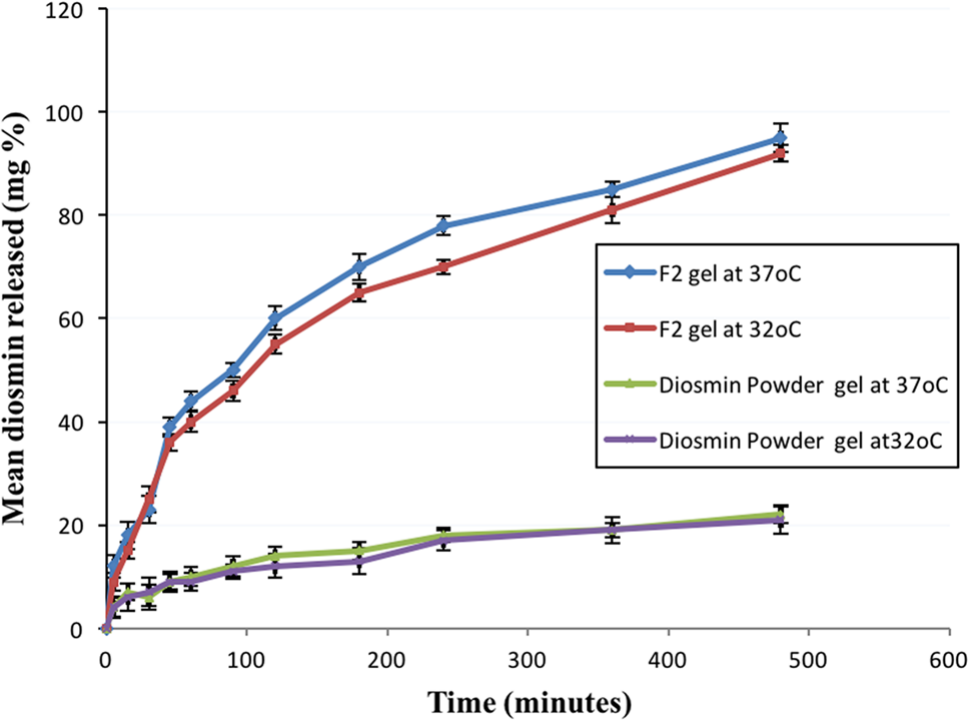
In-vitro release profile comparing release rate of gels at different temperatures 37 °C and 32 °C. Data presented as mean (of triplicates) ± SD

The formulated nanocrystal in a gel form was selected for the in-vivo study via topical route of administration. Un-expected release behavior might occur during diosmin release at skin temperature (32 °C). Therefore, further confirmation for the release study was done at 32 °C for the selected formulation in a form of a gel and diosmin powder gel. Furthermore, the amount of diosmin released from nanocrystal gel formulation after 45 min was decreased from 39 to 36% as the temperature decreased to 32 °C, Fig. 7. Therefore, only slight decrease in the drug release rate for the selected gel formulation and diosmin powder gel was observed when the temperature was decreased to 32 °C.

### Part 2

#### Psoriasis area severity index (PASI) assessment

**Fig. 8:**
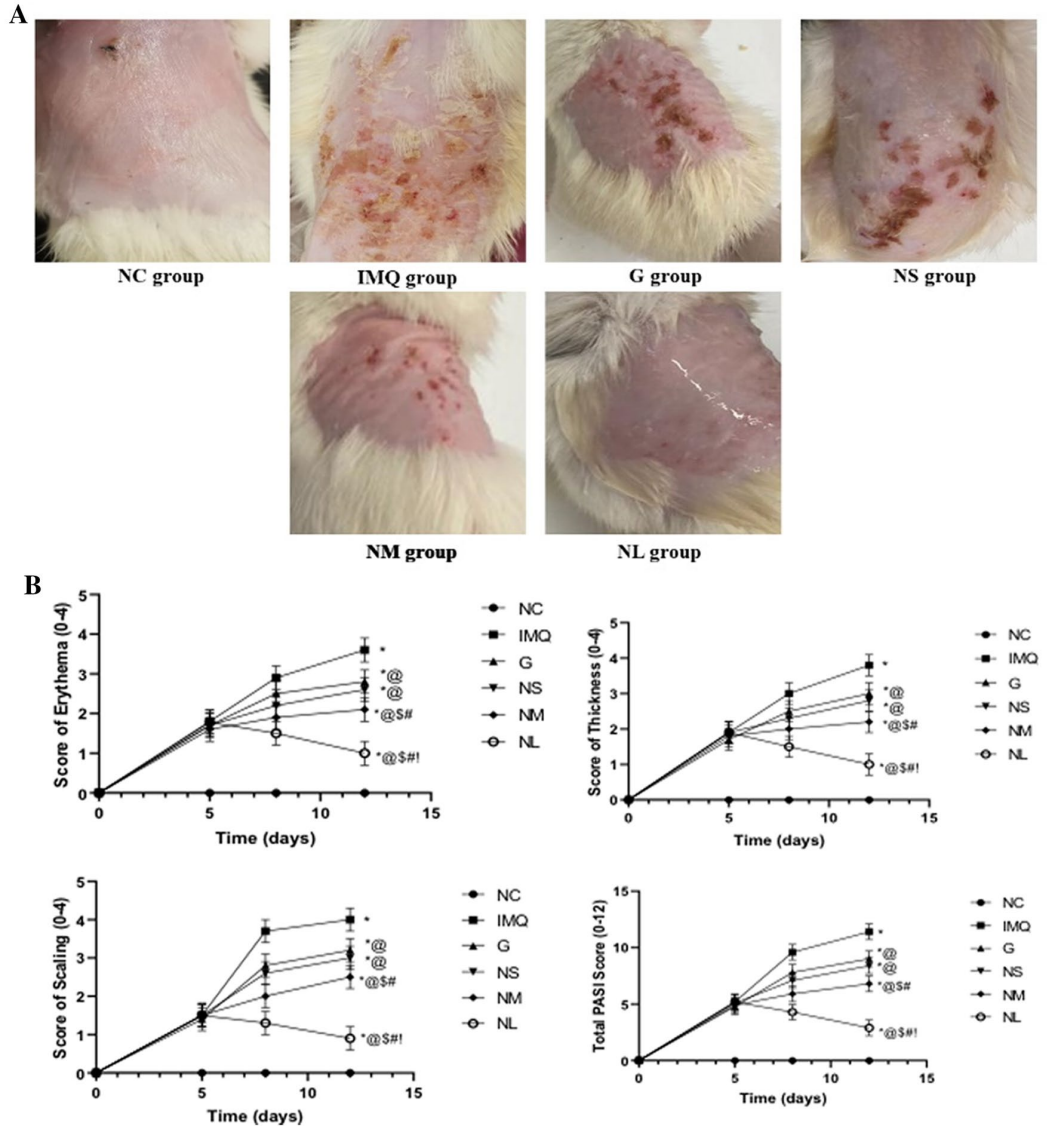
A Structural changes in the rat shaved dorsal skin regions following treatment of Imiquimod (IMQ) group with G: diosmin powder gel treated group at a dose of (9.6 mg/day), NS: Diosmin nanocrystal gel given at a dose of (4.8 mg/day), NM: Diosmin nanocrystal gel given at a dose of (9.6 mg/day), NL: Diosmin nanocrystal gel given at a dose of (19.2 mg/day) as compared to NC: normal control group on day 13 before sacrifice. B Changes in the Erythema, scaling, thickness, and PASI Scores following treatment of Imiquimod (IMQ) group with G: Diosmin powder gel treated group at a dose of (9.6 mg/day), NS: Diosmin nanocrystal gel given at a dose of (4.8 mg/day), NM: Diosmin nanocrystal gel given at a dose of (9.6 mg/day), NL: Diosmin nanocrystal gel given at a dose of (19.2 mg/day) as compared to NC: normal control group. Data presented as mean ± SD. *Significantly different from NC group. @Significantly different from IMQ group, *P* < 0.05. $ Significantly different from G group, *P* < 0.05. # Significantly different from NS group, *P* < 0.05.! Significantly different from NM group, *P* < 0.05, (Supplementary Table 2)

The structural changes in the rat shaved dorsal skin region in different groups as well as the PASI scores are shown, Fig. 8A, B. All PSAI parameters including erythema, scaling, and thickening were scored 0 on day 0 prior to IMQ application. All the aforementioned parameters were significantly elevated on day 5 as compared to day 0. The total PSAI score was significantly reduced after starting the diosmin various formulations with the most significant reduction observed on day 12 in the NL group reaching (2.9 ± 0.4) as compared to IMQ group (11.4 ± 2). Moreover, there was no significant difference between total PSAI score calculated on day 12 for G and NS treated groups.

#### Serum levels of inflammatory cytokines (IL-17 A, IL-22 and IL-23)

**Fig. 9:**
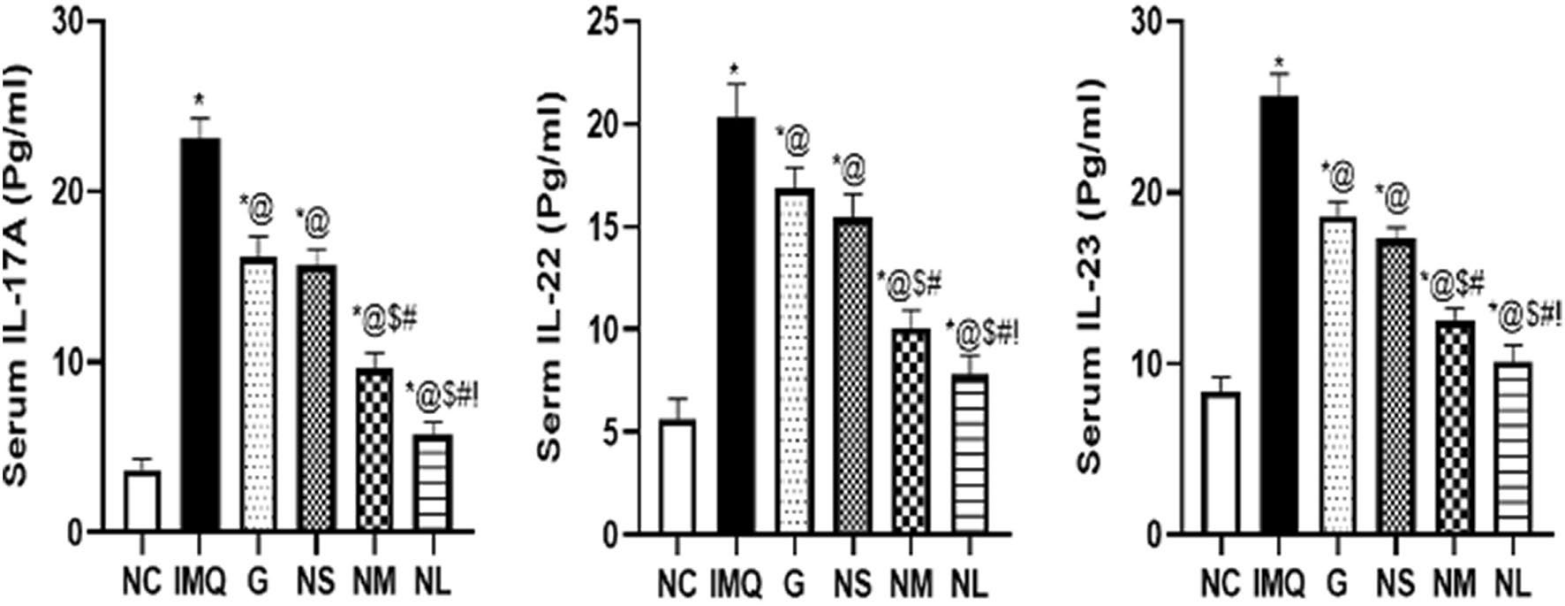
Changes in the serum inflammatory cytokines level following treatment of Imiquimod (IMQ) group with G: Diosmin powder gel treated group at a dose of (9.6 mg/day), NS: Diosmin nanocrystal gel given at a dose of (4.8 mg/day), NM: Diosmin nanocrystal gel given at a dose of (9.6 mg/day), NL: Diosmin nanocrystal gel given at a dose of (19.2 mg/day) as compared to NC: normal control group. Data presented as mean ± SD. *Significantly different from NC group. @Significantly different from IMQ group, *P* < 0.05. $ Significantly different from G group, *P* < 0.05. # Significantly different from NS group, *P* < 0.05.! Significantly different from NM group, *P* < 0.05, (Supplementary Table 2)

Figure 9, shows the change in the serum inflammatory cytokines level (IL-17A, IL-22 & IL-23) in different experimental groups. The serum IL-17A, IL-22, and IL-23 levels were markedly elevated in the IMQ treated group reaching (23.10 ± 1.20, 20.35 ± 1.61, and 25.58 ± 1.32 Pg/ml), respectively, as compared to the NC group approaching (3.72 ± 0.62, 5.66 ± 0.96, and 8.40 ± 0.82 Pg/ml), respectively. Upon the treatment with the different diosmin formulations, the most obvious significant declining in the serum IL-17A, IL-22 and IL-23 levels were observed in the NL treated group at a dose of (19.2 mg/day) reaching (5.76 ± 0.73, 7.80 ± 0.95, and 10.3 ± 0.98 Pg/ml), respectively**.** Furthermore, the serum inflammatory cytokine levels were significantly decreased in the G treated group at a dose of (9.6 mg/day) reaching (16.16 ± 1.2, 16.86 ± 1.02, and 18.64 ± 0.81 Pg/ml), respectively. The NM treated group at a dose of (9.6 mg/day/rat) showed a significant mitigation in the previously mentioned serum cytokines levels approaching (9.65 ± 0.91, 10.10 ± 0.84 and 12.55 ± 0.67 Pg/ml), respectively. Moreover, there was no significant difference between the effect of G treated group and NS treated group given at a dose of (4.8 mg/day) on the aforementioned inflammatory parameters. Finally, we could conclude the superior efficacy of the diosmin nanocrystal gel designed in the current study as compared to the diosmin powder gel formulation.

#### The TLR-7/8/ NF-kβ p-65, TNFAIP3, P-AKT/P-m-TOR/P70S6k. PCNA, and BCL-2 expression levels by western blot technique

**Fig. 10:**
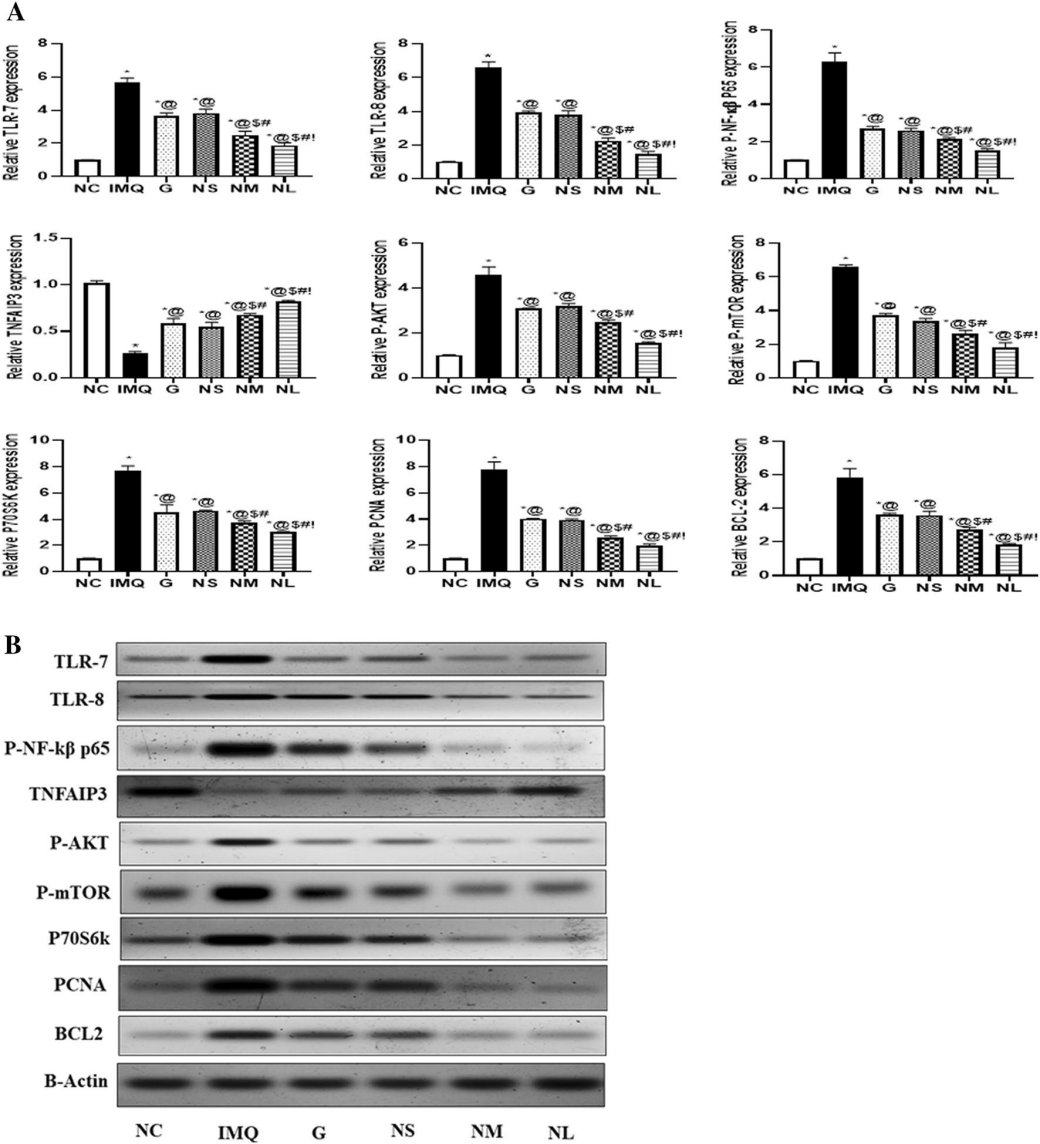
A/B Changes in TLR-7/8/ P-NF-kβ p-65, TNFAIP3, P-AKT/P-m-TOR/P70S6k. PCNA, and BCL-2 expression levels following treatment of Imiquimod (IMQ) group with G: Diosmin powder gel treated group at a dose of (9.6 mg/day), NS: Diosmin nanocrystal gel given at a dose of (4.8 mg/day), NM: Diosmin nanocrystal gel given at a dose of (9.6 mg/day), NL: Diosmin nanocrystal gel given at a dose of (19.2 mg/day) as compared to NC: normal control group. Data presented as mean ± SD. *Significantly different from NC group @Significantly different from IMQ group, *P* < 0.05. $ Significantly different from G group, *P* < 0.05. # Significantly different from NS group, *P* < 0.05.! Significantly different from NM group, *P* < 0.05, (Supplementary Table 2)

To reveal the role of immune pathways in psoriasis progression and treatment mechanisms, western blot analysis is performed to assess the protein expression of TLR-7/8/ P-NF-kβ p65, P-AKT/P-m-TOR/P70S6k, TNFAIP3, PCNA, and BCL-2, Fig. 10A, B. The TLR-7/8, P-NF-kβ p65, P-AKT, P-mTOR, and P70S6k expression were significantly elevated in the IMQ group highlighting their role in the disease immune pathogenesis. Where this elevation is notably reversed with various degrees in the different diosmin treated groups. The most significant reduction in the previously mentioned parameters was observed in the (NL) treated group at a dose of (19.2 mg/day). Moreover, the (G) treated group at a dose of (9.6 mg/day) caused the same level of mitigation in the TLR7/8, P-NF-kβ p65, P-AKT, P-mTOR, and P70S6k expression in the psoriatic skin lesions as that of the (NS) treated group at a dose of (4.8 mg/day).

On the other hand, TNFAIP3, an inhibitor of the NF-kβ pathway was shown to be significantly declined in the IMQ psoriasis induced group. Its expression was significantly restored upon the treatment with the different diosmin formulations with the greater efficacy of NL formulation.

To elucidate the effect of the diosmin formulation on the proliferation tendency of the keratinocytes in psoriatic skin tissues, western blot analysis was used to determine the expression levels of PCNA, and BCL2. The protein expression of the of the proliferation markers PCNA and the anti-apoptotic protein BCL2 was observed to be markedly increased in the IMQ psoriasis induced group, while their expression levels were significantly declined in all the treatment groups used in the current study. The comparative tackling of the PCNA, and BCL2 expression levels were in the order of NL > NM > NS.

#### Relative microRNA-31 expression level

**Fig. 11:**
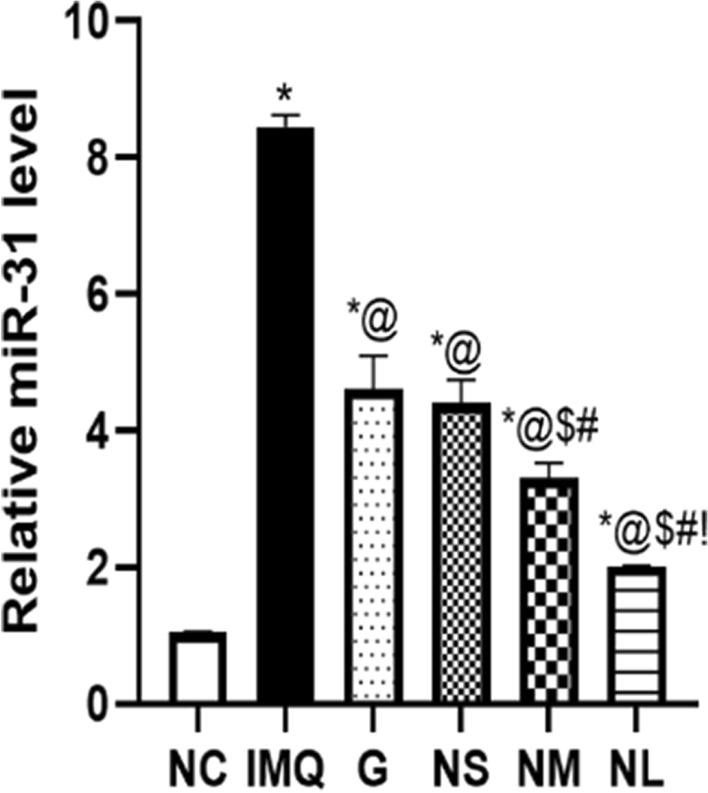
The relative miR-31 expression levels following treatment of Imiquimod (IMQ) group with G: diosmin powder gel treated group at a dose of (9.6 mg/day), NS: Diosmin nanocrystal gel given at a dose of (4.8 mg/day), NM: Diosmin nanocrystal gel given at a dose of (9.6 mg/day), NL: Diosmin nanocrystal gel given at a dose of (19.2 mg/day) as compared to NC: normal control group. Data presented as mean ± SD. *Significantly different from NC group @Significantly different from IMQ group, *P* < 0.05. $ Significantly different from G group, *P* < 0.05. # Significantly different from NS group, *P* < 0.05.! Significantly different from NM group, *P* < 0.05, (Supplementary Table 2)

The relative expression of miR-31 level was done by PCR technique to reveal the mechanisms of regulating the production of the inflammatory cytokines’ milieu, Fig. 11. The expression of miR-31 was observed to be upregulated in the IMQ group 7.5 folds compared to the NC group. Upon treatment with the different diosmin formulations (G, NS, NM and NL) significant decrease in the miR-31 expression was noticed by 46.5%, 48%, 61%, 64.5%, respectively.

#### Histopathological and immunohistochemical assessments

**Fig. 12:**
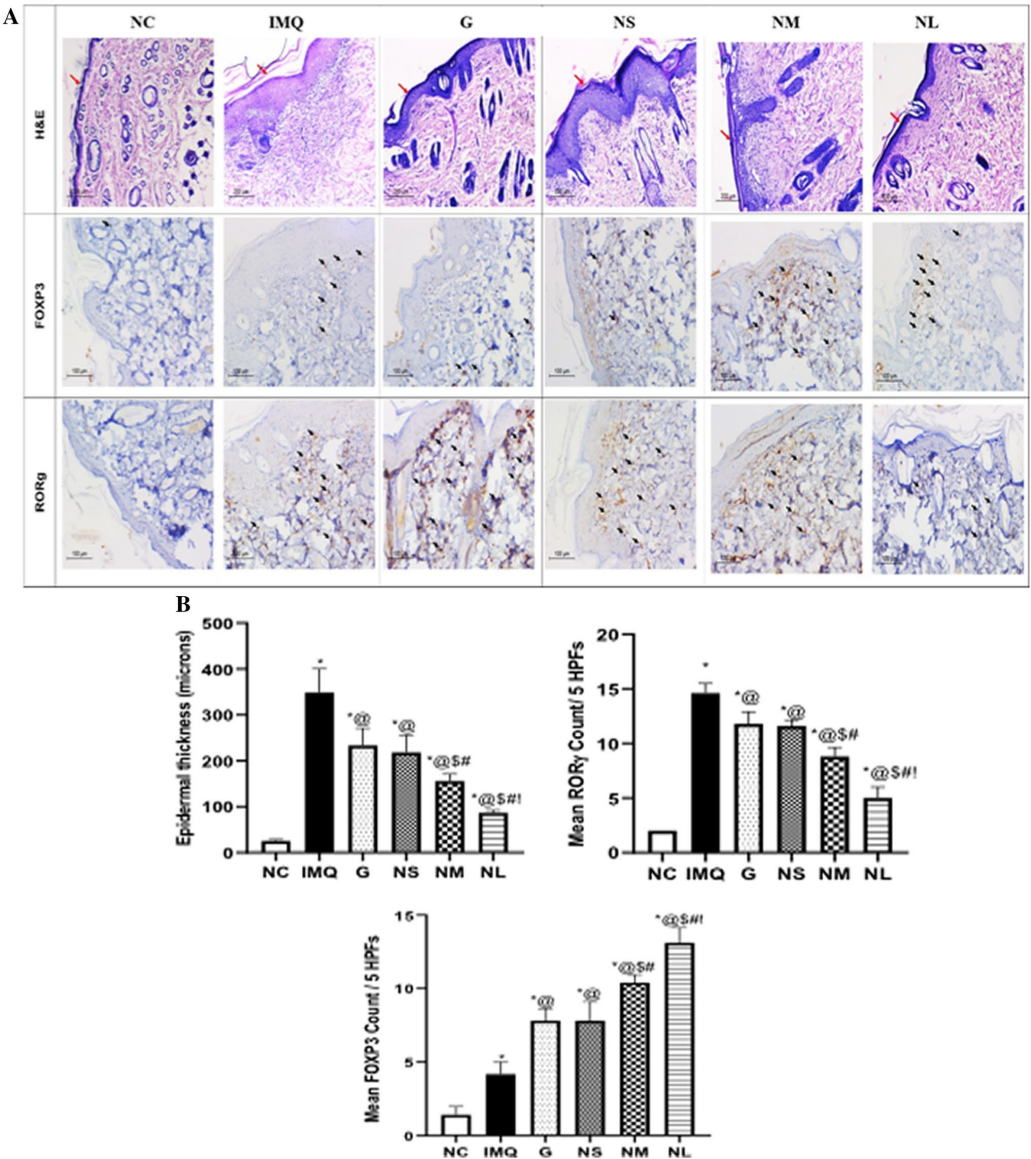
A Histopathological changes observed in H&E stained sections showing changes of epidermal thickness (red arrow denotes epidermis), (H&E, X100) as well as the immunohistochemical staining for FOXP3, and RORγ in skin (black arrows denote positive cells). (IHC, X200) following treatment of Imiquimod (IMQ) group with G: diosmin powder gel treated group at a dose of (9.6 mg/day), NS: Diosmin nanocrystal gel given at a dose of (4.8 mg/day), NM: Diosmin nanocrystal gel given at a dose of (9.6 mg/day), NL: Diosmin nanocrystal gel given at a dose of (19.2 mg/day) as compared to NC: normal control group. B Epidermal thickness (microns) as well as Th17-Treg balance within control and different treated groups. Data presented as mean ± SD. *Significantly different from NC group @Significantly different from IMQ group, *P* < 0.05. $ Significantly different from G group, *P* < 0.05. # Significantly different from NS group, *P* < 0.05.! Significantly different from NM group, *P* < 0.05, (Supplementary Table 2)

Histopathological assessment of H&E stain and immunohistochemical assessment of FOXP3 and RORγ in skin sections embedded in paraffin blocks from different experimental groups are illustrated in Fig. 12A, B.

The normal control group revealed thin epidermis composed of keratinized stratified squamous epithelium. The dermis was loose collagenous. No significant dermal inflammation was seen. The IMQ treated model showed evident pathologic changes. The epidermis was significantly thickened reaching (348.2 microns ± 52.24) as compared to NC group reaching (25.29 microns ± 3.399). Hyper and Parakeratosis was seen as well as elongated rete ridges. Upper dermis showed moderate inflammatory infiltrate and congested dermal vessels. In Drug treated groups histologic findings varied greatly from IMQ treated model. The G treated groups which showed mild improvement of epidermal thickness (233.2 microns ± 37.69). Best anti-psoriatic effect was seen in groups received large dose where epidermal thickness decreased to be (86.31 microns ± 5.913) compared to (155.4 microns ± 15.3) and (218.2 microns ± 37.43) in medium NM and NS treated groups, respectively.

Regarding immunostaining done to reveal the balance between Th17-Treg cells infiltrated within the skin tissue, negative control group showed occasionally detected FOXP3 dermal lymphocytes while few RORγ positive cells were seen.

In IMQ treated model, FOXP3 positive T lymphocytes in dermal inflammatory cells were minimal (3–5/5HPFs). They increased with various degrees in different diosmin treated groups. They reached (7–9/5HPFs) in the G treated group. Furthermore, they increased markedly after treatment with a large dose of diosmin nanocrystal gel (NL) approaching (12–14/5HPFs). This was significantly higher than their count in both medium (NM) and small dose (NS) groups (10–11/5HPFs) and (8–9/5HPFs) respectively.

Meanwhile, RORγ positive cells were frequently detected in IMQ treated model reaching (14–16/5HPFs). They declined in the different treated groups in various doses and formulations. In the G treated group their count reached (Yan et al. 2015; Xu et al. 2013; Mercurio et al. 2021). The least count was seen when in the NL treated group reaching (4–6/5HPFs) followed by medium dose used in the NM treated group approaching (8–10/5HPFs). While the effect seen in NS treated group, revealed numerous RORγ positive cells approaching (11–12/5HPFs) and this effect was not significantly different from that shown in the G treated group.

#### Statistical correlation

The Statistical correlation was done to reveal the dynamic equilibrium between Treg & T helper 17. It showed a significant negative correlation between Foxp3 & ROR γ in the IMQ group, where the over-expression of RORγ is accompanied with down regulation of Foxp3. The balance is gradually restored within the treatment groups till the significant negative correlation is observed in the NL group indicating the efficacy of the diosmin formulation in inhibiting ROR γ expression resulting in upregulation of Foxp3, Table 4. In addition, statistical correlations revealed that RORγ expression is positively correlated with inflammatory cytokines (IL-17A, IL-22 & IL-23) production and epidermal thickness, while on the other hand, a significant negative correlation is observed between FOXP3 expression and inflammatory cytokines as well as epidermal thickness, Table 5.

**Table 4:** Statistical correlation between FOXP3 and RORγ among different experimental groups

Group	Spearman rho coefficient
IMQ	*r*_*s*_ = − 0.41039, *p* (2-tailed) = 0.49254
G	*r*_*s*_ = − 0.6455, *p* (2-tailed) = 0.23944
NS	*r*_*s*_ = − 0.25, *p* (2-tailed) = 0.68504
NM	*r*_*s*_ = 0.41248, *p* (2-tailed) = 0.49011
NL	*r*_*s*_ = − 0.75, *p* (2-tailed) = 0.14429

*IMQ* Imiquimod group, *G* diosmin powder gel treated group at a dose of (9.6 mg/day), *NS* Diosmin nanocrystal gel given at a dose of (4.8 mg/day), *NM* Diosmin nanocrystal gel given at a dose of (9.6 mg/day), and *NL* Diosmin nanocrystal gel given at a dose of (19.2 mg/day)

**Table 5:** Statistical correlation between FOXP3, RORγ within NL group with inflammatory cytokines and epidermal thickness

	FOXP3	RORγ
IL17A	*r*_*s*_ = − 0.59761, *p* (2-tailed) = 0.2103	*r*_*s*_ = 0.52463, *p* (2-tailed) = 0.28525
IL-23	*r*_*s*_ = − 0.92582, *p* (2-tailed) = 0.00805	*r*_*s*_ = 0.58635, *p* (2-tailed) = 0.22127
IL-22	*r*_*s*_ = − 0.80238, *p* (2-tailed) = 0.05472	*r*_*s*_ = 0.37033, *p* (2-tailed) = 0.4699
Epidermal thickness	*r*_*s*_ = − 0.53229, *p* (2-tailed) = 0.27697	*r*_*s*_ = 0.57926, *p* (2-tailed) = 0.2283

## Discussion

### Part 1

The results of the current study revealed the efficacy of Diosmin nano-formulations as a potential therapy of psoriasis. Diosmin nanocrystals were prepared using anti-solvent precipitation technique. The delayed appearance of aggregates after increasing the concentration of HPMC might be attributed to the insufficient amount of stabilizer failed to attain complete coverage of drug molecules which is required to maintain repulsion between particles in nanocrystal. Sufficient amount of stabilizer led to formation of a hydrodynamic boundary layer providing a steric hindrance to agglomeration and crystal growth. (Gericke et al. 2020). The increase in the particle size after increasing the concentration of poloxamer 407 could be attributed to the fact that above the critical micelle concentration (cmc) the surfactant molecules tend to be oriented in a micelle rather than to be adsorbed on the surface of nanocrystals^.^ (Perumal et al. 2022; Jin et al. 2021).

Furthermore, the decrease in particle size with the increase of MC concentration might be attributed to MC adsorption at the solid liquid interface reducing the surface tension and leading to an increased rate of nucleation as it will be accumulated in the hydrodynamic layer between the particles, hindering their collision and subsequent growth. However, using MC in a higher concentration led to formation of highly viscous solution that hindered the formulation of nanocrystals. (Abdellatif et al. 2021). Thus it could be concluded that, cellulose polymers provided more steric stabilization than poloxamer due to the fact that, Poloxamer is a nonionic block copolymer of polyoxyethylene polyoxypropylene–polyoxyethylene (PEO–PPO–PEO), where the hydrophobic PPO part adsorb onto the particle surface with the PEO chains extending into the solution. The reason for the relatively low adsorption observed compared to cellulose polymers, could be due to the fact that the methyl groups of the PPO chain do not render the chain sufficiently hydrophobic to adsorb to the very hydrophobic particle surface. (Mfoafo et al. 2022).

When polymers used in concentrations above critical flocculation concentration (CFC) (similar to cmc in surfactants), polymer molecules initiate depletion or bridging flocculation of the particles in suspension. Also, at increased concentration of polymer, there will be an increase in osmotic pressure leads to increased attraction among colloidal particles leading to agglomeration. (Khelfa et al. 2021). Moreover, diosmin needle-shaped crystals were observed over time and more obvious when nanocrystals were kept at room temperature. Increasing temperature promotes the increase of Ostwald ripening phenomenon. Smaller crystals possess a relatively higher saturation solubility compared to larger particles. This could result in a concentration gradient and, hence the drug diffuses from the more concentrated solution around the smaller particle to the surrounding of the larger crystals leading to supersaturation in the surrounding of these large crystals and subsequently to crystallization of molecules on the crystal surface. (Gommes 2019).

The saturation solubility of diosmin in ortho phosphate buffer and borate buffer pH 9 is very low, so achieving release experiment using dialysis bag method in this medium was very challenging. (Mujtaba et al. 2020). The % diffused from all formulations did not exceed 10% in three hours. Similarly, pH 10 caused also difficulties in discrimination between formulations, as the percent diffused not exceed 25% in 24 h. So dialysis bag method was not suitable for non-sink condition. Therefore, using non-diffusion system (Dissolution USP type II apparatus) was conducted at pH10. The saturation solubility in borate buffer pH 10 was about four folds higher than in pH 9. Thus, it was selected as a discriminating medium for the current study. Therefore, using either HPMC or MC in optimum concentration provided high system stabilization.

### Part 2

The main immune hallmark of psoriasis is the dominance of the inflammatory environment including the activation of both innate and adaptive arms of the immune system. This inflammatory milieu includes the over activation of dendritic cells, macrophages, T helper lymphocytes in addition to the dysregulated balance between Treg and Th17 cells leading to cytokine mediated chronic inflammation and uncontrolled keratinocyte proliferation (Lowes et al. 2014). Imiquimod-induced psoriasis animal model is considered a reliable experimental model for studying the immune hallmarks of psoriasis and resembles the complex nature of the skin inflammatory microenvironment of the disease (Jabeen et al. 2020).

Many previous studies used imiquimod to induce effective psoriasis model in mice (Sakai et al. 2016; Li et al. 2021). While in the current study a successful rat model was established with application of 125 mg of 5% IMQ that contain 62.25 mg IMQ topically on the the shaved skin on the back of rats for 5 consecutive days with ideal disease manifestations, including keratinocytes hyperproliferation, elevation of inflammatory markers and disrupted innate and adaptive immune systems balance (Smajlović et al. 2021).

Imiquimod is considered a TLR7/8 agonist, which can induce and intensify psoriasis in rat model via the activation of IL-23/IL-17A axis (Tahir et al. 2013). TLR7/8 are expressed on plasmacytoid and myeloid dendritic cells, respectively. Their over activation leads to increase in the production of interferon-γ (IFN-ɤ) thus enhancing the autoreactive T cells population. This engagement of the adaptive immune cells’ population will in turn exacerbates the psoriasis manifestations (Flutter and Nestle 2013). Antagonist of TLR7/8 previously shown their efficacy by inhibiting Th1/Th17 responses and inflammatory cytokines production (Jiang et al. 2013). Additionally, it has been reported that upregulated TLR7/8 in IMQ group can subsequently activate the known transcription factor NF-κB to increase the production of inflammatory cytokines and activation of IL-23/IL-17 axis (Li et al. 2013; Bender et al. 2020).

These previously mentioned studies were in line with our results that showed obvious elevation in the expression of TLR7/8, P-NF-κB P65 and elevation in the serum inflammatory cytokines (IL-17A, IL-22, IL23) in IMQ group. Moreover, diosmin nanocrystal gel in the highest dose (19.2 mg/day) used in the study caused the most significant downregulation in the expression of TLR7/8 with subsequent inhibition in the activation of NF-κB and this lead the most obvious reduction in the serum inflammatory cytokines level. Additionally, it was mentioned previously that activation of NF-κB can increase the transcription of anti-apoptotic protein (BCL2), inhibiting apoptosis of skin cells in psoriasis. This finding was similar to our results as they revealed increase in the expression of NF-κB as well as BCL2 in the skin of IMQ treated rats (Mercurio et al. 2021).

In addition, our results showed that the expression of (TNFAIP3/A20) which is known inhibitor of NF-κB was mitigated in the skin of psoriatic rats, and this was supported by previous studies (Devos et al. 2019; Sahlol et al. 2019). Its expression was mostly elevated in the NL treated group. This reveals the role of diosmin nanocrystal gel in increasing the expression of TNFAIP3/A20 inhibiting the activation of NF-κB and inflammatory milieu.

There was a significant increase in the miR-31 level in the skin of IMQ treated rats which was confirmed by another studies (Yan et al. 2015; Xu et al. 2013). Moreover, Diosmin different formulations decreased the miR-31 level with the most significant reduction observed in the NL treated group. It has been reported that activated NF-κB in psoriasis can induce miR-31 which subsequently can suppress STK40 to aid in further activation of NF-κB creating a positive feedback loop between miR-31 and NF-κB. This emphasizes the role of miR-31 level in psoriatic lesions that can augment skin inflammation (Yan et al. 2015; Xu et al. 2013).

In this study there was a significant elevation in the expression level of P-AKT, P-mTOR, and P70S6K in the skin of IMQ group. It was obvious that the diosmin nanocrystals were more effective than diosmin powder gel in suppressing the expression of the aforementioned proteins expression in the skin of psoriatic rats. It has been reported that intracellular signaling pathway AKT/m-TOR/P70S6K was activated in the skin of psoriatic patients and in skin of mice treated with Imiquimod leading to uncontrolled epidermal cells proliferation (Mercurio et al. 2021; Karagianni et al. 2022). Additionally, our results revealed significant elevation in the expression of PCNA in the skin of untreated psoriatic rats, indicating an increased level of cell proliferation. Previous studies demonstrated the effect of diosmin of different proliferation markers in different types of cancers such as hepatocellular carcinoma (Tahir et al. 2013), oral squamous cell carcinoma (Osman et al. 2022), colon (Zeya et al. 2022) and gastric cancer (Hu et al. 2013). Moreover, AKT indirectly induces the transcription of anti-apoptotic genes such as BCL2 via NF-κB factors (Mercurio et al. 2021; Ozes et al. 1999; Kane et al. 1999).

The role of adaptive immune cells such as T helper cells including Th17 and their balance with Treg lymphocytes is considered an essential factor for psoriasis pathogenesis and progression. Where the role of FOXP3 regulatory T cells in inhibiting Th1 and Th17 cells is well established to be crucial for immune homeostasis, inhibition of inflammatory cytokines production and prevention of autoimmunity (Stockenhuber et al. 2018). Different studies reported a contradicted results regarding the abundance of FOXP3+ Tregs in psoriasis, some studies reported the decrease in the number of Tregs in psoriatic skin lesion and patients’ peripheral blood, while others showed that there is no difference in circulating Treg frequency. The current study revealed a significant negative correlation between the level of Th17 and Treg within the studied groups, supporting that the abundance of Th17 in the IMQ induction group is accompanied by a decrease in the Treg levels (rs = − 0.41039, p (2-tailed) = 0.49254). and upon treatment of different diosmin formulations the Treg/Th17 balance is restored with the highest efficacy in the NL formulation.

The plasticity between the phenotypes of Treg and Th17 is previously reported, where a portion of Tregs were seemed to be transformed to Th17-like phenotype in the presences of inflammatory cytokines such as IL1, IL6 or IL17. This transformation is accompanied by loop activation of cytokine production and maintaining the inflammatory environment resulting in the exaggeration of psoriasis symptom (Bovenschen et al. 2011).

That controlled interaction between TLR7/8 expressing antigen presenting cells, FOXP3+ Tregs and RORγ+ Th17 is strongly correlated with the inflammatory cytokines milieu and psoriatic symptoms represented by the overactivation of keratinocyte proliferation and epidermal thickness. Previous studies supported the finding of the importance of the IL17/IL22/IL23 axis in the pathogenesis of psoriasis, where IL23 is reported not only to support the Th17 lymphocyte producing IL-17 (McGeachy et al. 2009), but also stimulate IL-17 production via innate T lymphocyte such as Gamma delta T lymphocytes (Tγδ) (Qi et al. 2021). In addition, different studies highlighted the vital role of IL22 in psoriasis induction, its over expression in the psoriatic lesions, and its correlation with immune cells imbalance and disease severity (Hao 2014). Considering the forementioned findings, the IL17/IL22/IL23 axis could be considered a reliable biomarker for monitoring psoriasis progression and treatment efficacy.

The studies done to evaluate the effect of diosmin on inflammatory immune axis are considered scarce. Imam et al. study on the effect of diosmin on T cells and pro inflammatory cytokines production in lung injury supports our findings. Where the abovementioned study reported the efficacy of diosmin in reducing CD4+ total T cells population as well as IL2, IL6 and IL17 production thus confirming the immunomodulatory anti-inflammatory properties of diosmin (Imam et al. 2015).

The results of the current study showed that diosmin nano-formulas have the ability to significantly decrease the epidermal thickness within the drug treated groups with the highest efficacy noticed in the diosmin large dose formulation. The current study is considered the first study to investigate the efficacy of different diosmin formulations to halt the immune pathogenesis and inflammatory environment of psoriasis. Previous studies reported the anti-inflammatory and immunomodulatory effect of diosmin on the skin keratinocytes in other dermatological diseases such as atopic dermatitis via modulation of Th2 cells activity and the inflammatory cytokines such as IL6 and IL1 (Osman et al. 2022; Feldo et al. 2022). In addition, Li et al. recently showed that hesperidin, the citrus flavonoid that diosmin is derived from, has the ability to reduce the psoriatic like skin lesions in mice and inhibit the keratinocytes proliferation (Lee et al. 2021).

## Conclusion

According to the above-mentioned results obtained from the current study, it is suggested that diosmin nanocrystal gel formulations can be a promising treatment for psoriatic lesions due to its ability to halt the immunopathogenesis of psoriasis, via modulating TLR7,8/NF-κB/micro RNA-31, AKT/mTOR/P70S6K milieu and Tregs/Th17 balance. In addition to inhibiting inflammatory cytokines production and producing remarkable improvement in the skin epidermal thickness.

## Supplementary Information

Below is the link to the electronic supplementary material.

Supplementary file1 (DOCX 1539 KB)

Supplementary file2 (JPG 1044 KB)

Supplementary file3 (JPG 1217 KB)

Supplementary file4 (JPG 1106 KB)

Supplementary file5 (JPG 1238 KB)

Supplementary file6 (JPG 1220 KB)

Supplementary file7 (JPG 1160 KB)

Supplementary file8 (JPG 1366 KB)

Supplementary file9 (JPG 901 KB)

Supplementary file10 (JPG 1140 KB)

Supplementary file11 (JPG 917 KB)

Supplementary file12 (DOCX 52 KB)

## Data Availability

All data generated or analyzed during this study are included in this published article.
