# Supplementary material for: Diosmin nanocrystal gel alleviates imiquimod-induced psoriasis in rats via modulating TLR7,8/NF-κB/micro RNA-31, AKT/mTOR/P70S6K milieu, and Tregs/Th17 balance
Source: Inflammopharmacology. 2023 Apr 3;31(3):1341–59. doi: 10.1007/s10787-023-01198-w (PMC10229696; doi:10.1007/s10787-023-01198-w)
Supplement: Supplementary file 1 — Supplementary file1 (DOCX 1539 KB) [file 10787_2023_1198_MOESM1_ESM.docx]

**Table : Light microscope images of different diosmin nanocrystal formulations stored at 25^o^C and 4^o^C (magnified400X).**

| Diosmin nanocrystals  (freshly prepared) | | Room temperature  25^o^C | Refrigerator  4^o^C |
| --- | --- | --- | --- |
| **F1**  **(Diosmin :HPMC E15)**  **(1:0.5)**  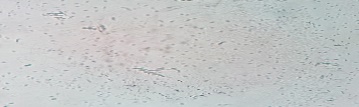  **P.S: 350.40 nm**  **PDI: 0.62** | **Day 2** | 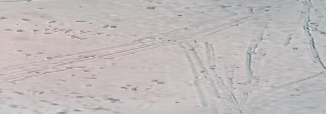 | 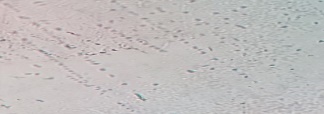 |
|  | **Day 5** | 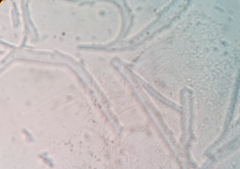 | 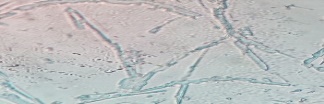 |
| **F2**  **(Diosmin:HPMC E15)**  **(1:1)**  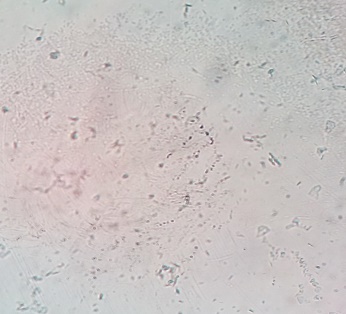  **P.S: 276.90 nm**  **PDI: 0.43** | **Day 2** | 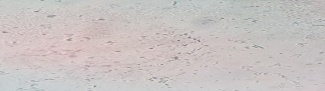 | 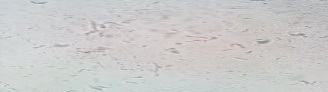 |
|  | **Day 5** | 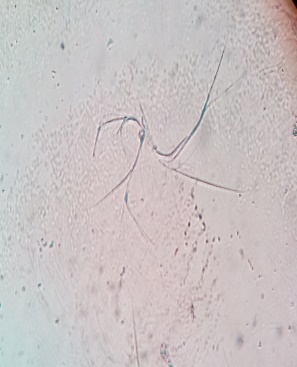 | 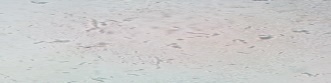 |
|  | **Day 7** | 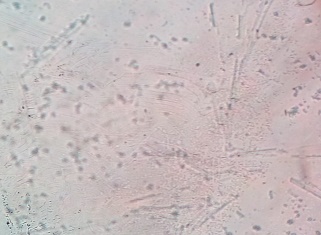 | 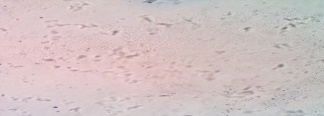 |
| Diosmin nanocrystals  (freshly prepared) | | Room temperature  25^o^C | Refrigerator  4^o^C |
| **F3**  **(Diosmin:HPMC E15)**  **(1:2)**  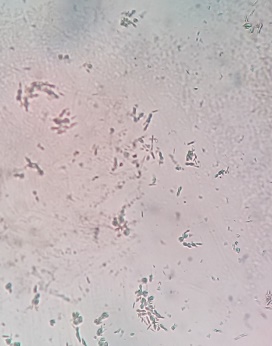  **P.S: 257.00 nm**  **PDI: 0.62** | **Day 2** | 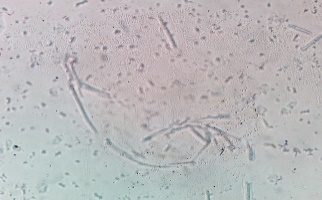 | 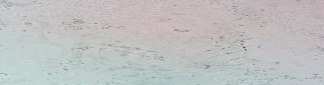 |
|  | **Day 5** | 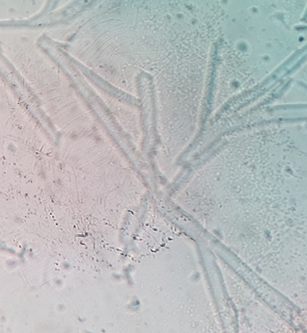 | 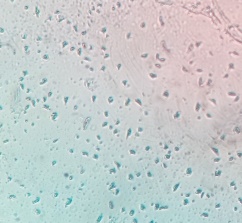 |
|  | **Day 7** | 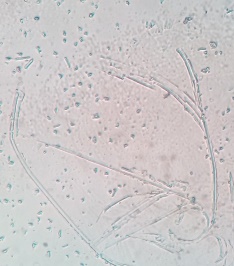 | 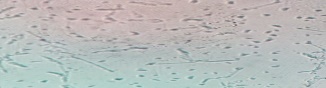 |
| **F4(Diosmin:Poloxamer 407)(1:0.5)**  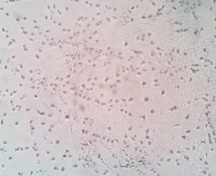  **P.S: 473.4 nm**  **PDI: 0.700** | **Day 2** | 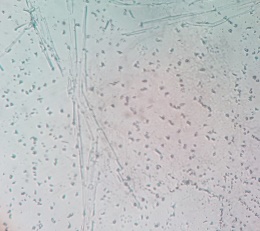 | 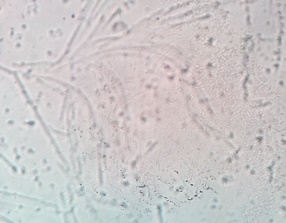 |
| **F5**  **(Diosmin:Poloxamer 407)(1:1)**  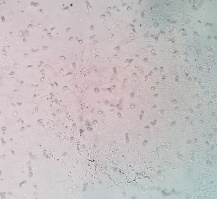  **P.S: 436.90 nm**  **PDI: 0.73** | **Day 2** | 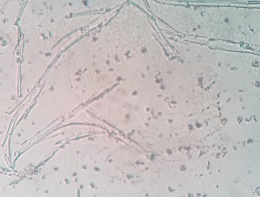 | 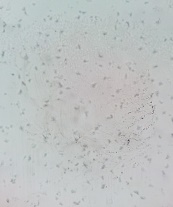 |
|  | **Day 5** | 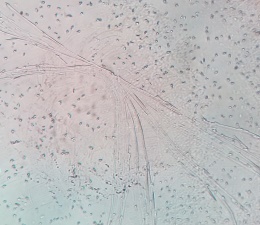 | 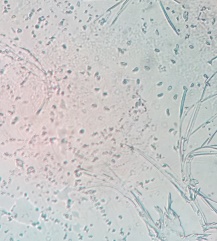 |
| Diosmin nanocrystals  (freshly prepared) | | Room temperature  25^o^C | Refrigerator  4^o^C |
| **F6**  **(Diosmin:Poloxamer407)**  **(1:2)**    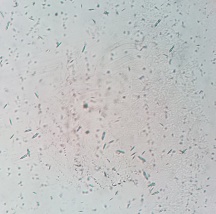  **P.S: 567.70 nm**  **PDI: 0.68** | **Day 2** | 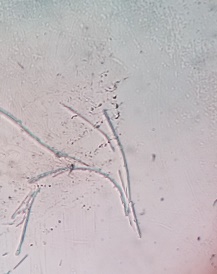 | 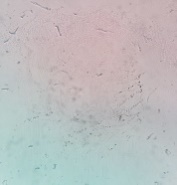 |
|  | **Day 5** | 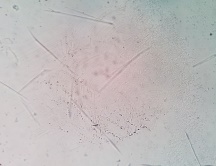 | 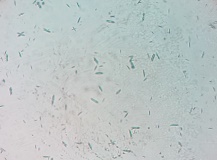 |
|  | **Day 7** | 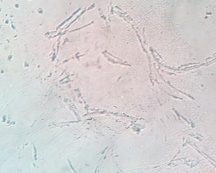 | 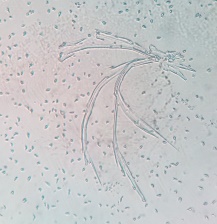 |
| **F7**  **(Diosmin:MC)**  **(1:0.5)**  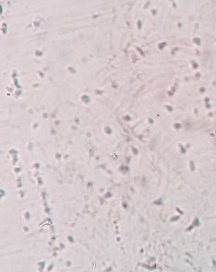  **P.S: 347.40 nm**  **PDI: 0.47** | **Day 2** | 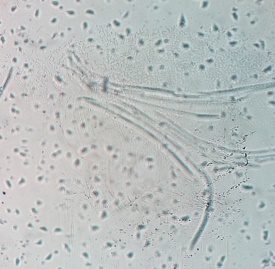 | 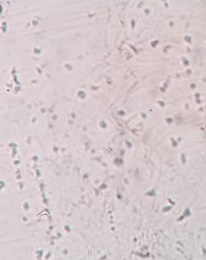 |
|  | **Day 5** | 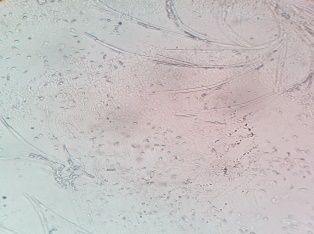 | 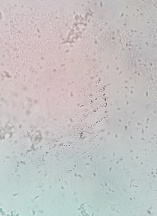 |
|  | **Day 7** | 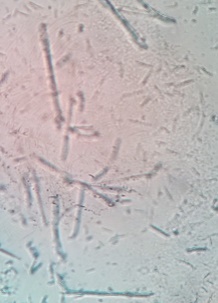 | 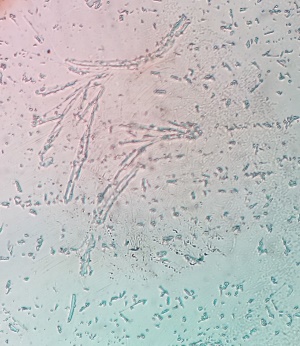 |
| Diosmin nanocrystals  (freshly prepared) | | Room temperature  25^o^C | Refrigerator  4^o^C |
| **F8**  **(Diosmin:MC)**  **(1:1)**  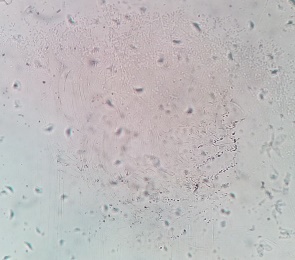  **P.S: 295.80 nm**  **PDI: 0.44** | **Day 2** | 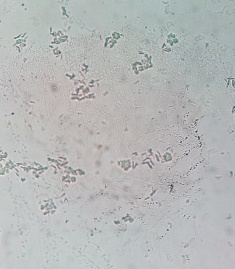 | 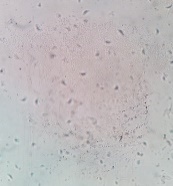 |
|  | **Day 5** | 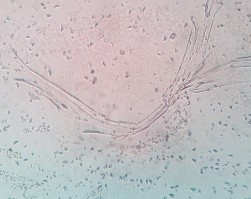 | 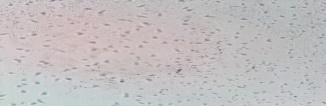 |
|  | **Day 7** | 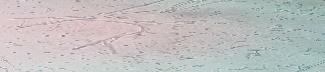 | 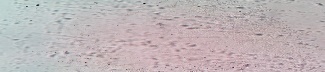 |
| **F9**  **(Diosmin:HPMC E15:Poloxamer 407)**  **(1:0.5:0.5)**  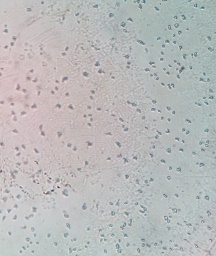  **P.S: 266.60 nm**  **PDI: 0.59** | **Day 2** | 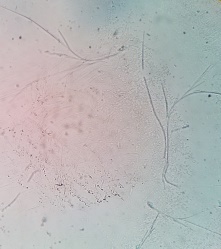 | 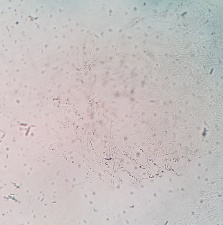 |
|  | **Day 5** | 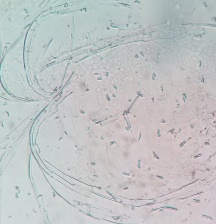 | 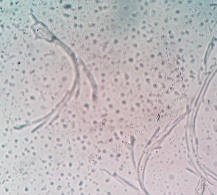 |
| Diosmin nanocrystals  (freshly prepared) | | Room temperature  25^o^C | Refrigerator  4^o^C |
| **F10**  **(Diosmin:HPMC E15:Poloxamer 407)**  **(1:1:1)**  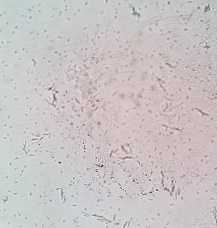  **P.S: 301.10 nm**  **PDI: 0.64** | **Day 2** | 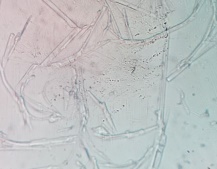 | 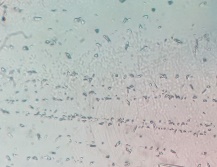 |
|  | **Day 5** | 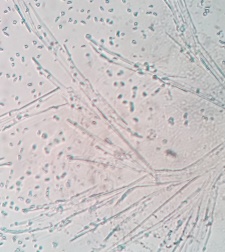 | 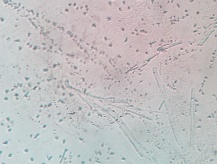 |
| **F11**  **(Diosmin:MC:**  **Poloxamer 407)**  **(1:1:1)**  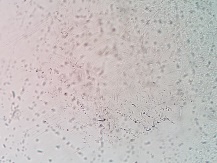  **P.S: 474.50 nm**  **PDI:0.53** | **Day 2** | 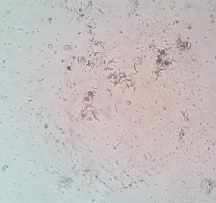 | 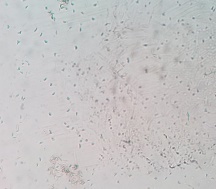 |
|  | **Day 5** | 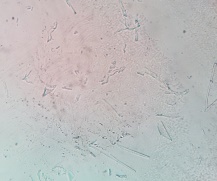 | 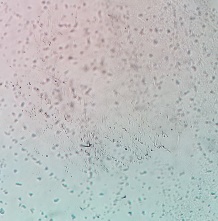 |
|  | **Day 7** | 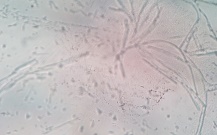 | 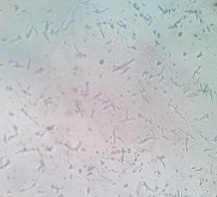 |
