# Supplementary figures and images for: Diosmin nanocrystal gel alleviates imiquimod-induced psoriasis in rats via modulating TLR7,8/NF-κB/micro RNA-31, AKT/mTOR/P70S6K milieu, and Tregs/Th17 balance
Source: Inflammopharmacology. 2023 Apr 3;31(3):1341–59. doi: 10.1007/s10787-023-01198-w (PMC10229696; doi:10.1007/s10787-023-01198-w)

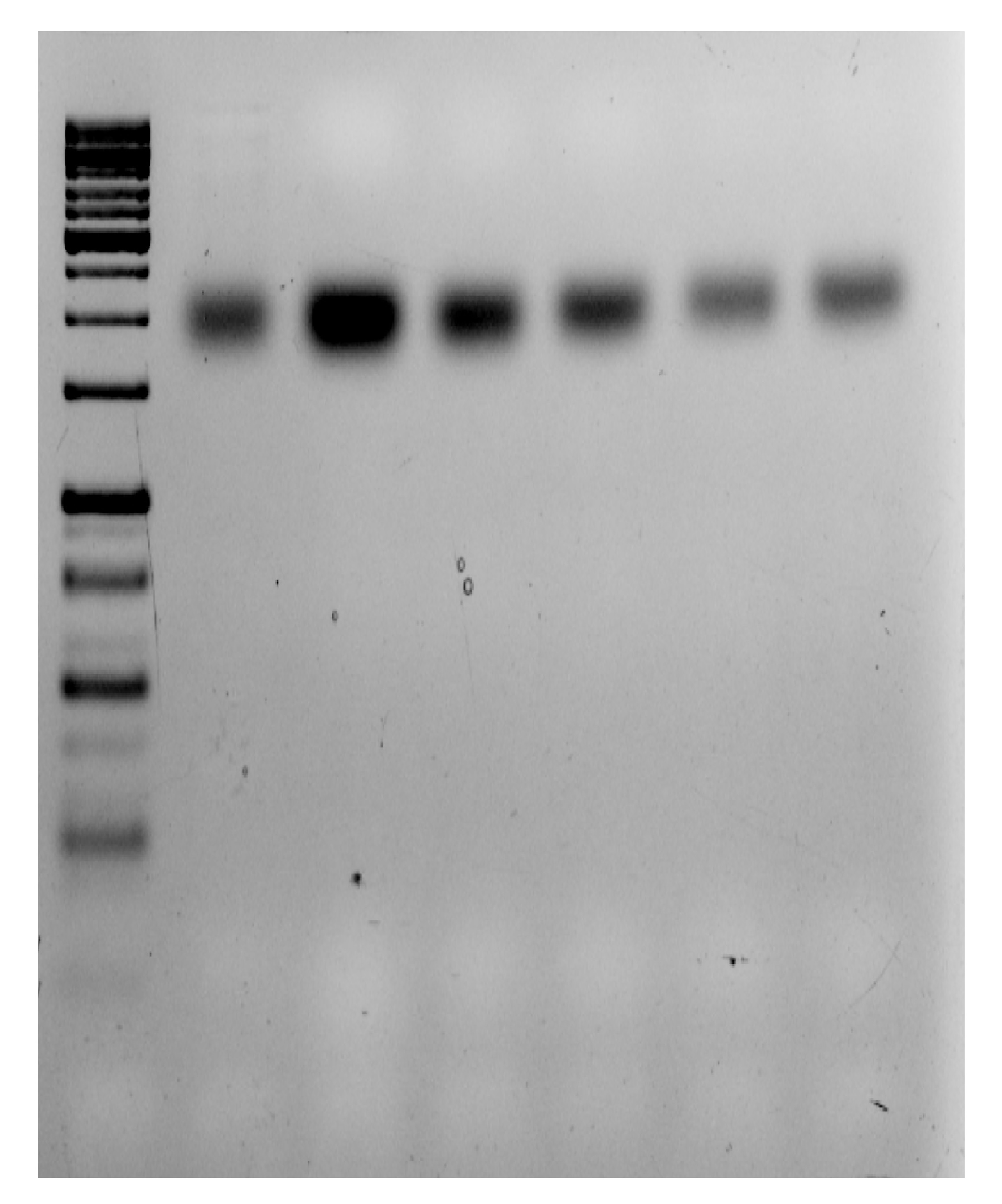

Supplement: Supplementary file 2 — Supplementary file2 (JPG 1044 KB) [file 10787_2023_1198_MOESM2_ESM.jpg]

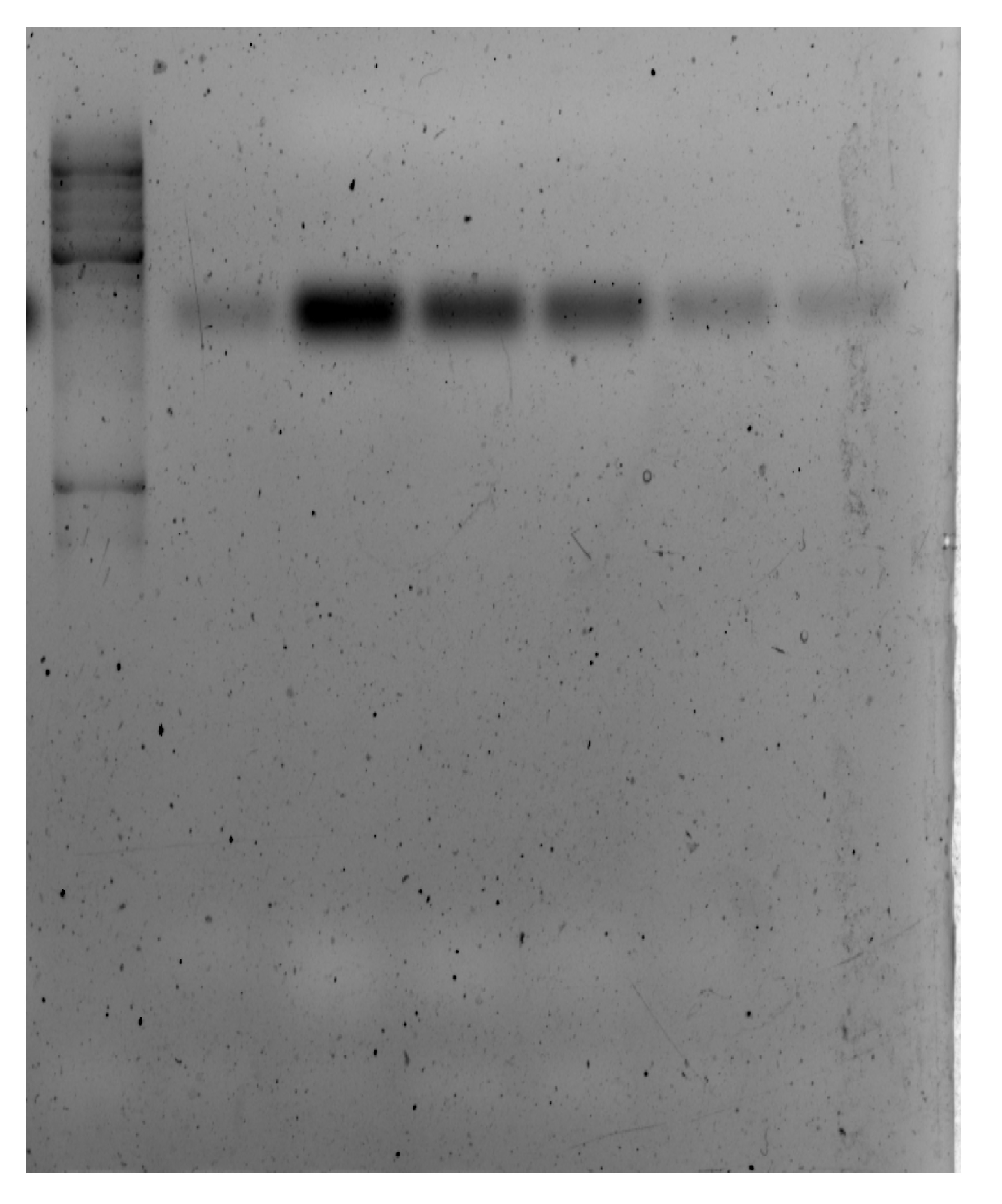

Supplement: Supplementary file 3 — Supplementary file3 (JPG 1217 KB) [file 10787_2023_1198_MOESM3_ESM.jpg]

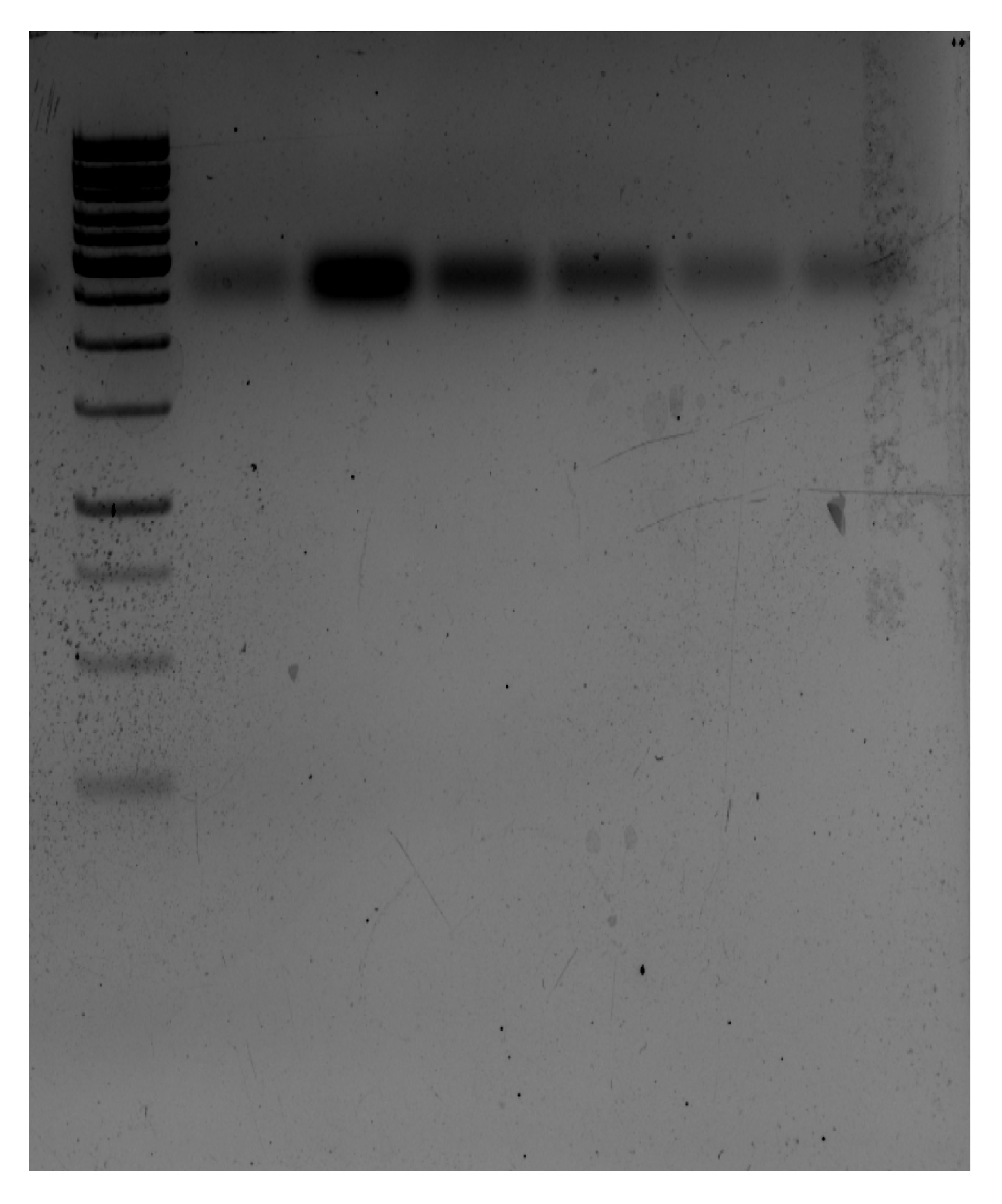

Supplement: Supplementary file 4 — Supplementary file4 (JPG 1106 KB) [file 10787_2023_1198_MOESM4_ESM.jpg]

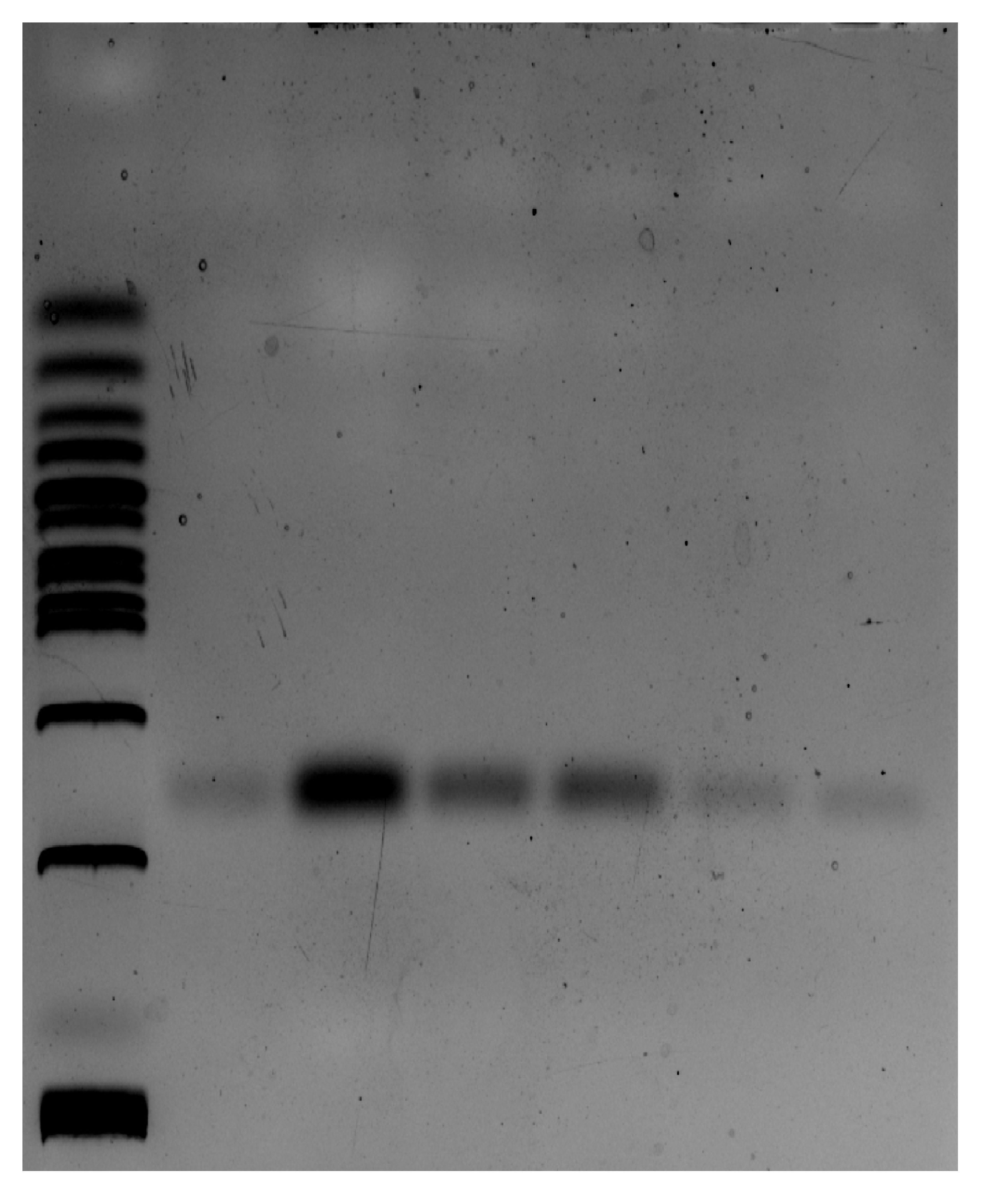

Supplement: Supplementary file 5 — Supplementary file5 (JPG 1238 KB) [file 10787_2023_1198_MOESM5_ESM.jpg]

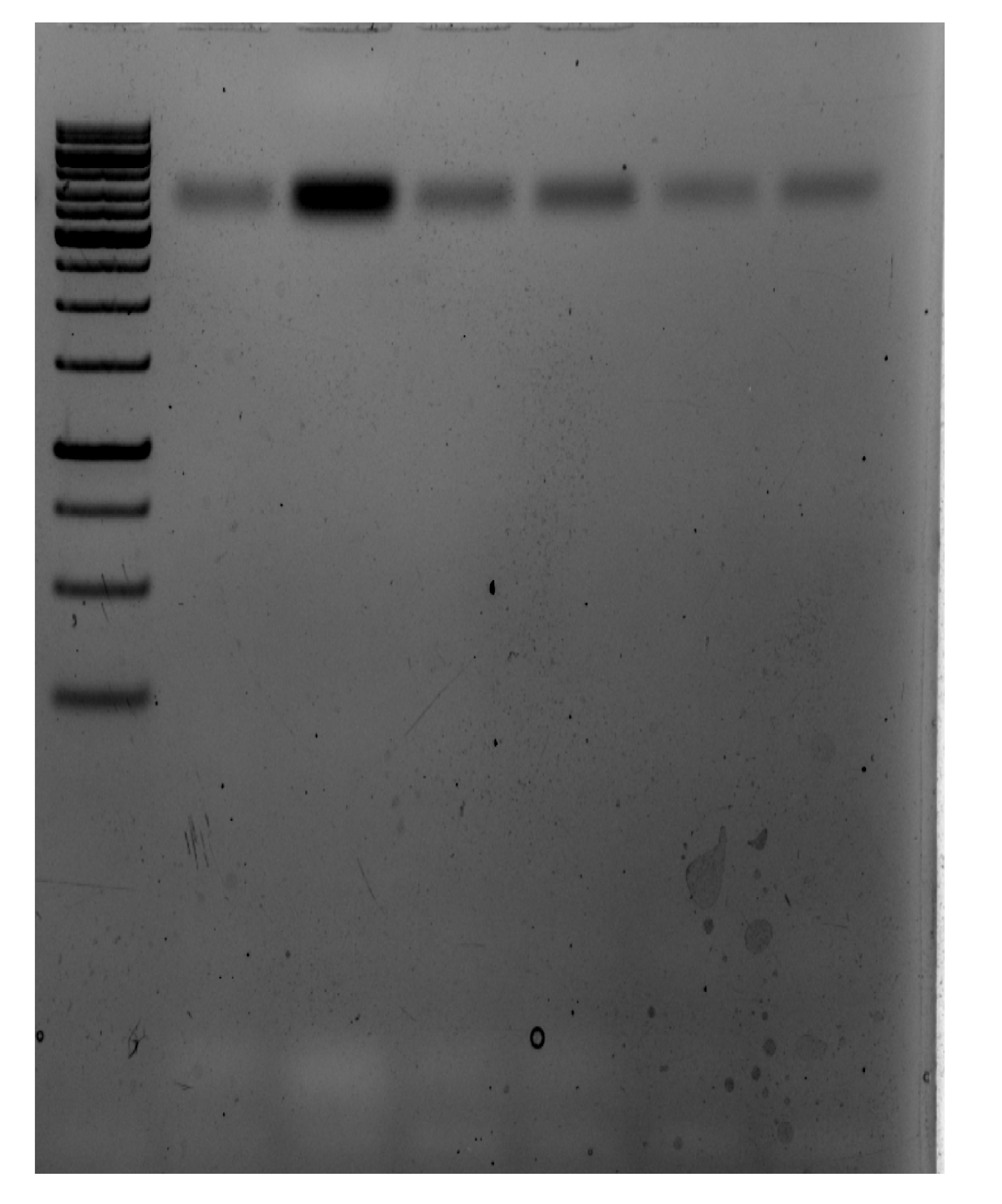

Supplement: Supplementary file 6 — Supplementary file6 (JPG 1220 KB) [file 10787_2023_1198_MOESM6_ESM.jpg]

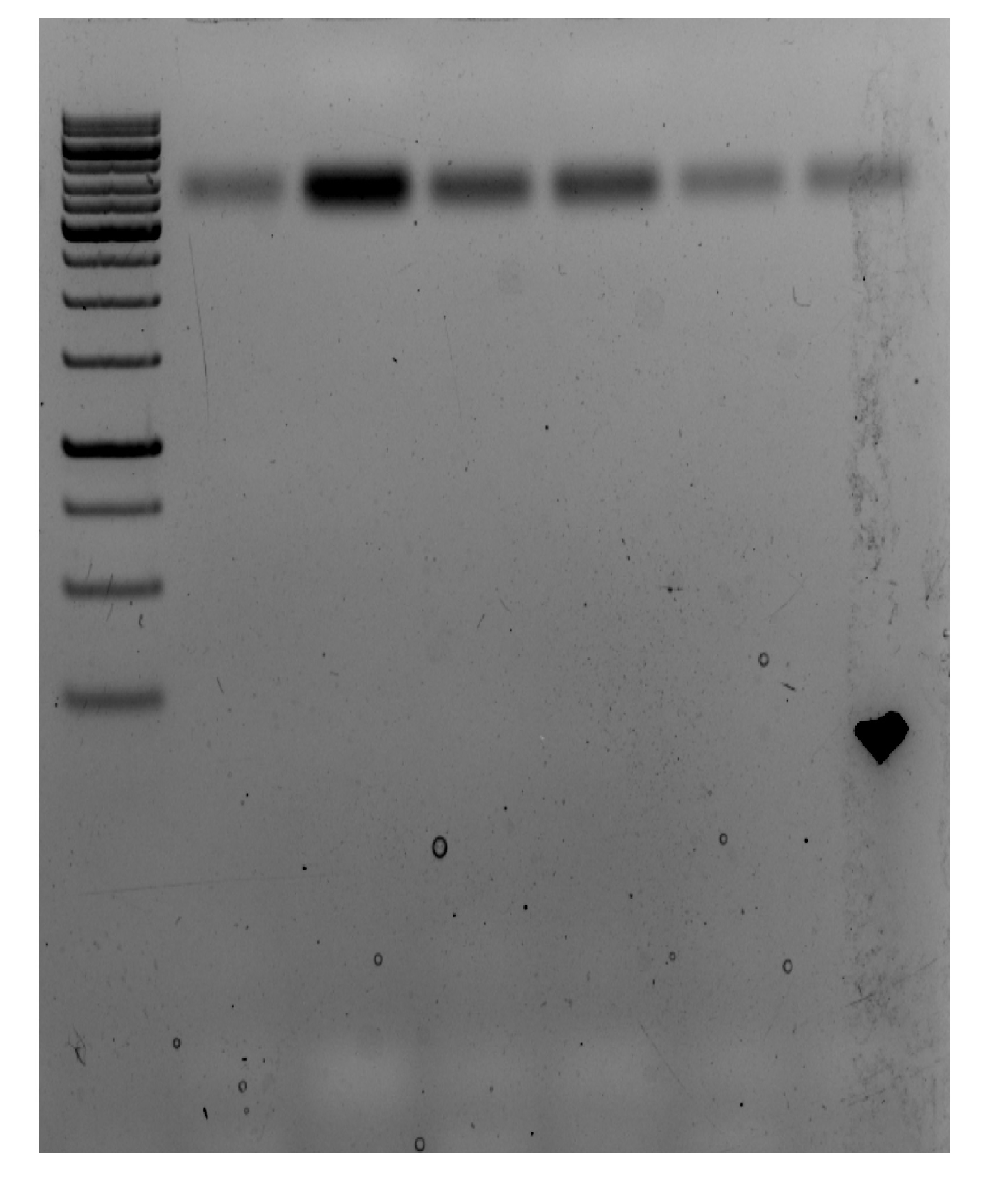

Supplement: Supplementary file 7 — Supplementary file7 (JPG 1160 KB) [file 10787_2023_1198_MOESM7_ESM.jpg]

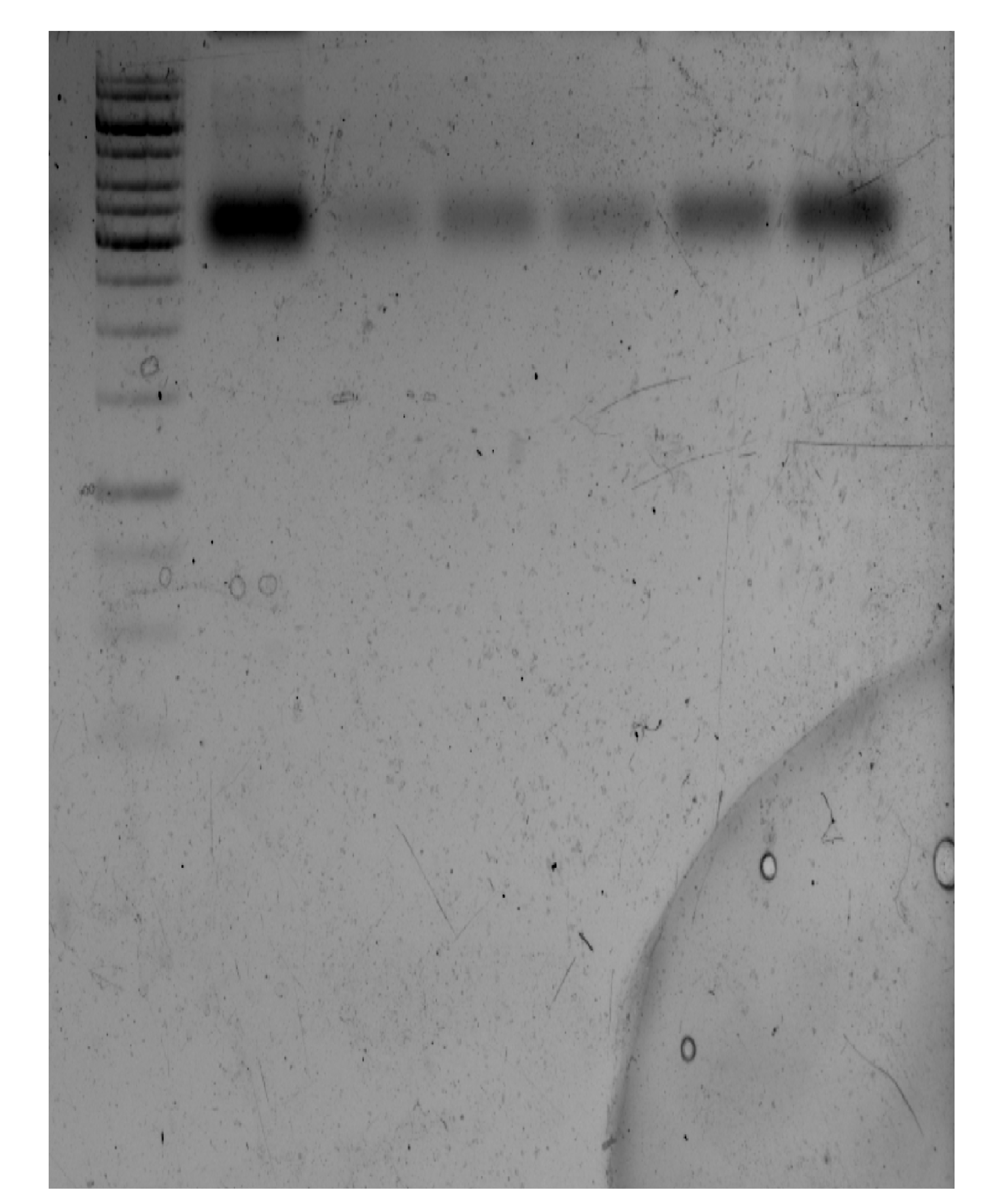

Supplement: Supplementary file 8 — Supplementary file8 (JPG 1366 KB) [file 10787_2023_1198_MOESM8_ESM.jpg]

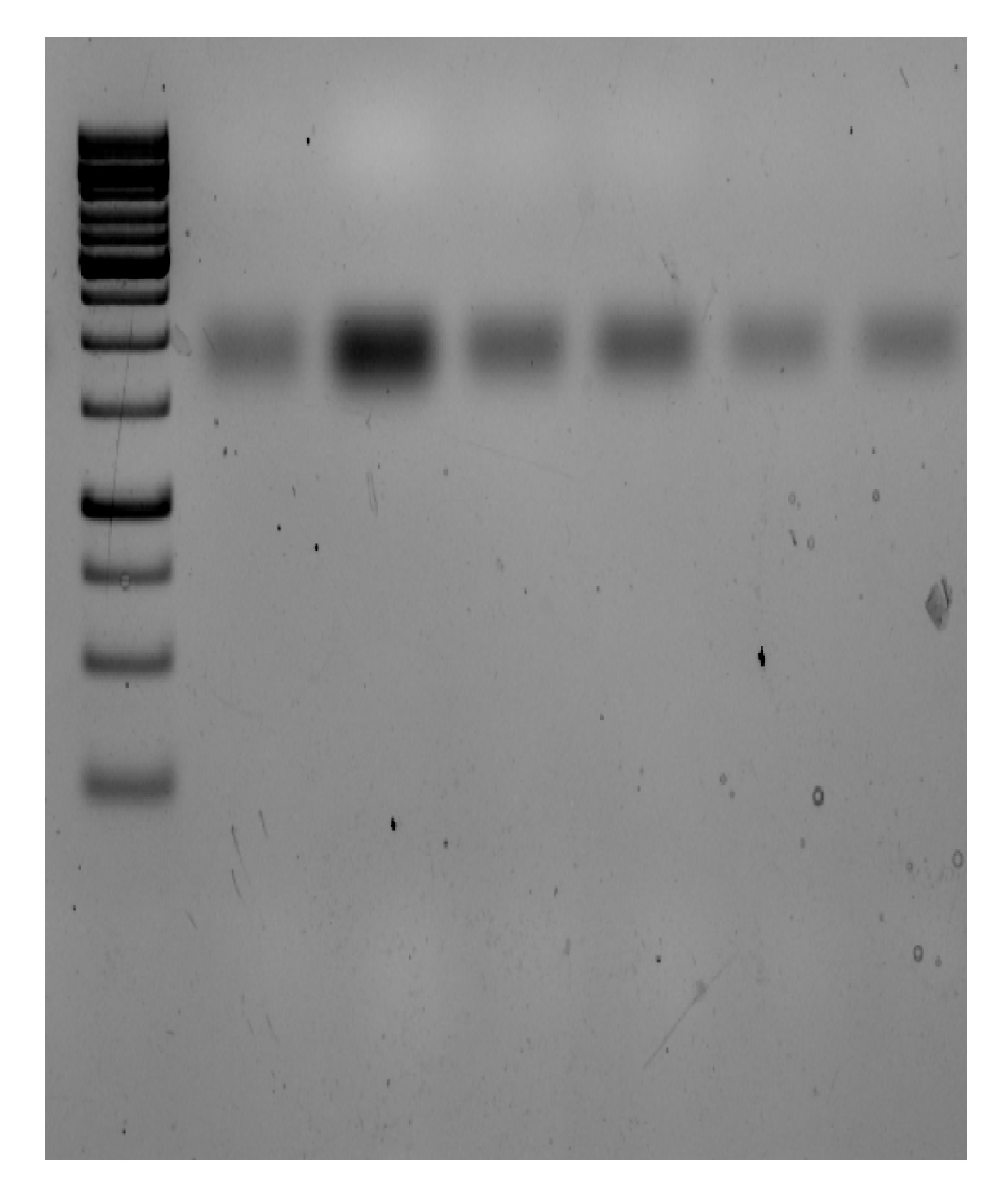

Supplement: Supplementary file 9 — Supplementary file9 (JPG 901 KB) [file 10787_2023_1198_MOESM9_ESM.jpg]

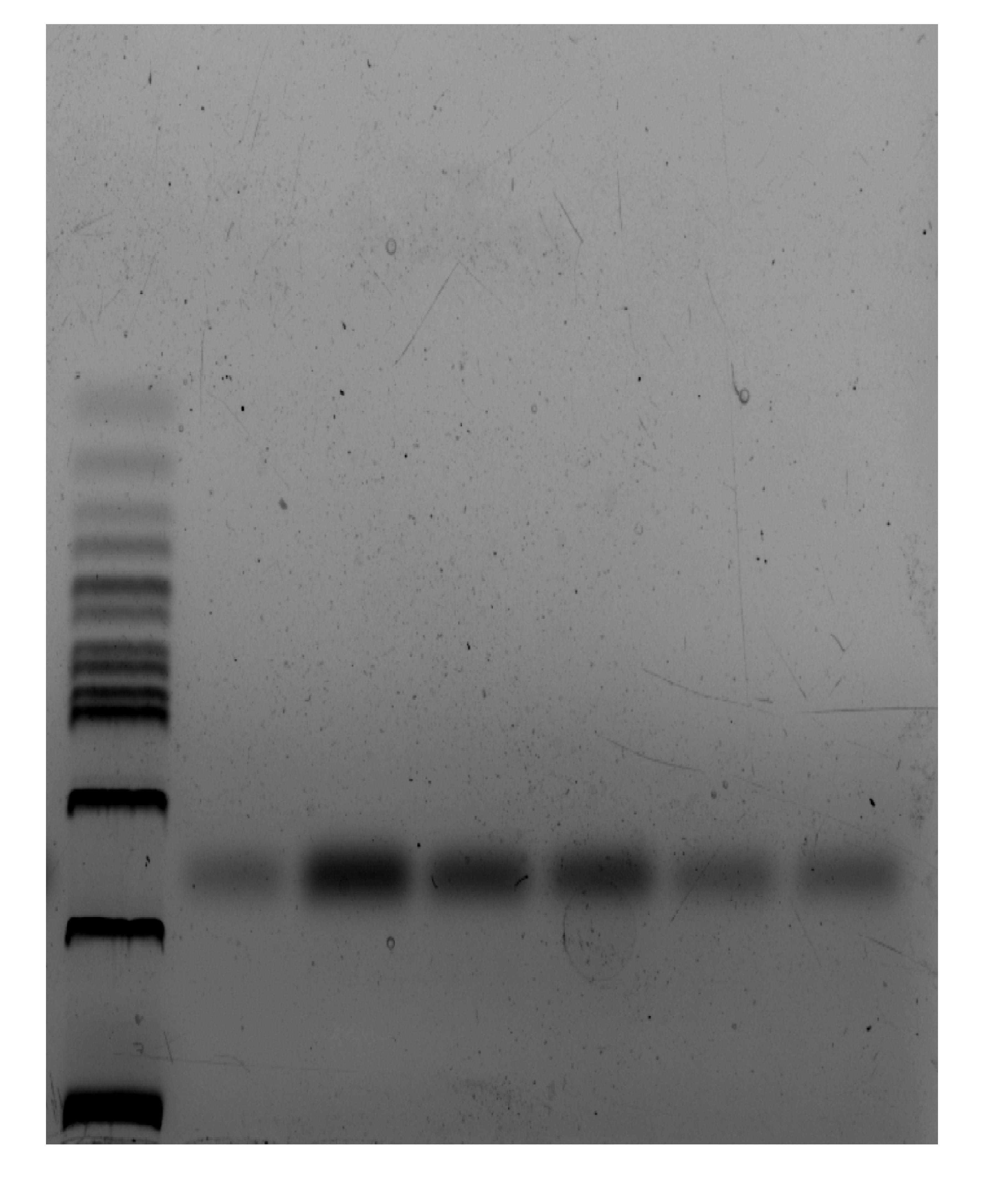

Supplement: Supplementary file 10 — Supplementary file10 (JPG 1140 KB) [file 10787_2023_1198_MOESM10_ESM.jpg]

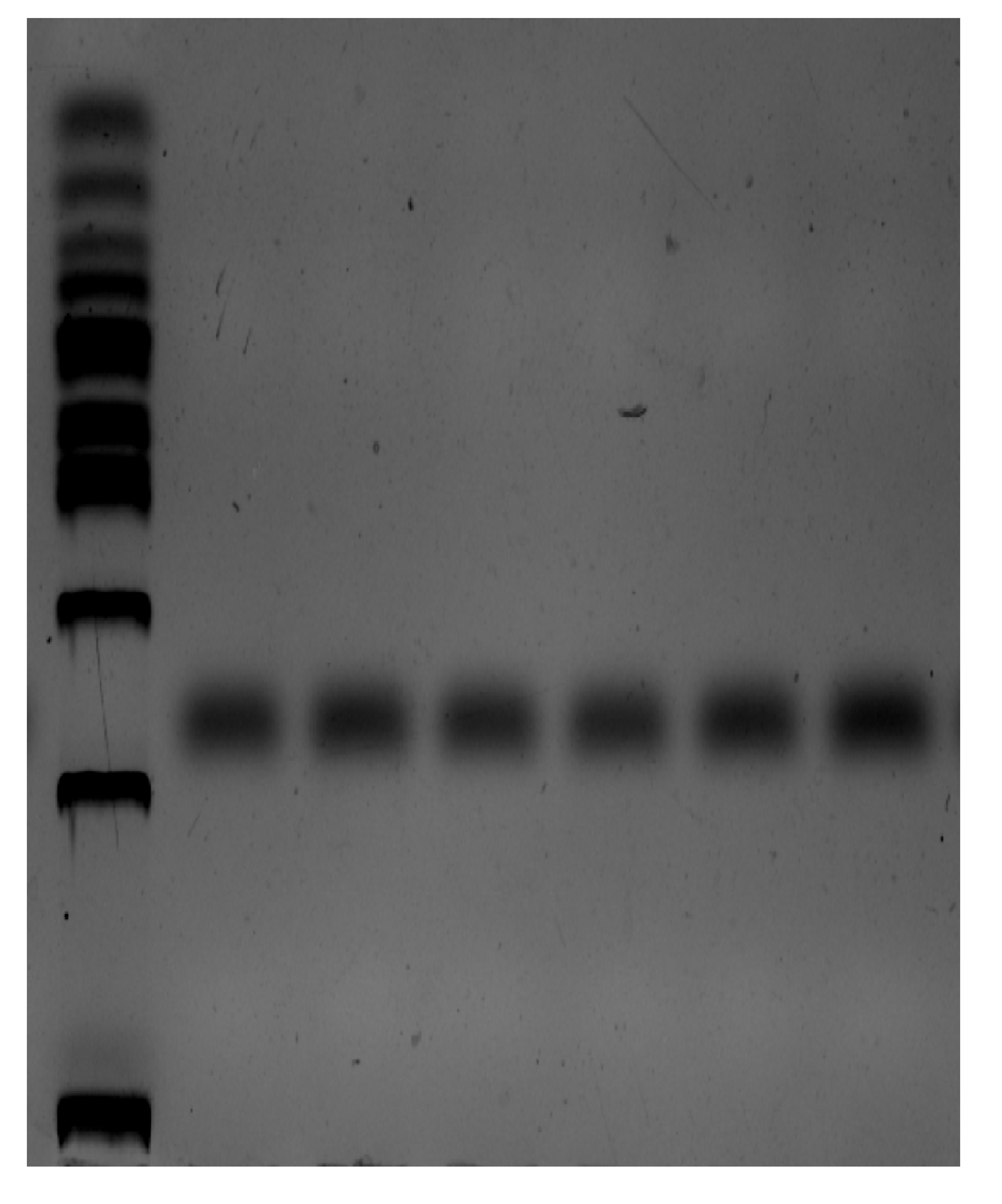

Supplement: Supplementary file 11 — Supplementary file11 (JPG 917 KB) [file 10787_2023_1198_MOESM11_ESM.jpg]
