# Supplementary material for: Diosmin nanocrystal gel alleviates imiquimod-induced psoriasis in rats via modulating TLR7,8/NF-κB/micro RNA-31, AKT/mTOR/P70S6K milieu, and Tregs/Th17 balance
Source: Inflammopharmacology. 2023 Apr 3;31(3):1341–59. doi: 10.1007/s10787-023-01198-w (PMC10229696; doi:10.1007/s10787-023-01198-w)
Supplement: Supplementary file 12 — Supplementary file12 (DOCX 52 KB) [file 10787_2023_1198_MOESM12_ESM.docx]

**Supplementary material**

**Supplementary table 1: serum level of IL 17A**

| Tukey's multiple comparisons test | Mean Diff. | 95.00% CI of diff. | Significant? | Summary | Adjusted P Value |
| --- | --- | --- | --- | --- | --- |
| NC vs. IMQ | -19.37 | -21.06 to -17.68 | Yes | **** | <0.0001 |
| NC vs. G | -12.43 | -14.12 to -10.74 | Yes | **** | <0.0001 |
| NC vs. NS | -11.94 | -13.63 to -10.25 | Yes | **** | <0.0001 |
| NC vs. NM | -5.932 | -7.625 to -4.239 | Yes | **** | <0.0001 |
| NC vs. NL | -2.037 | -3.730 to -0.3435 | Yes | * | 0.0113 |
| IMQ vs. G | 6.940 | 5.247 to 8.633 | Yes | **** | <0.0001 |
| IMQ vs. NS | 7.432 | 5.739 to 9.125 | Yes | **** | <0.0001 |
| IMQ vs. NM | 13.44 | 11.75 to 15.13 | Yes | **** | <0.0001 |
| IMQ vs. NL | 17.34 | 15.64 to 19.03 | Yes | **** | <0.0001 |
| G vs. NS | 0.4917 | -1.201 to 2.185 | No | ns | 0.9476 |
| G vs. NM | 6.500 | 4.807 to 8.193 | Yes | **** | <0.0001 |
| G vs. NL | 10.40 | 8.702 to 12.09 | Yes | **** | <0.0001 |
| NS vs. NM | 6.008 | 4.315 to 7.701 | Yes | **** | <0.0001 |
| NS vs. NL | 9.903 | 8.210 to 11.60 | Yes | **** | <0.0001 |
| NM vs. NL | 3.895 | 2.202 to 5.588 | Yes | **** | <0.0001 |

**Supplementary table 2: serum level of IL 22**

| Tukey's multiple comparisons test | Mean Diff. | 95.00% CI of diff. | Significant? | Summary | Adjusted P Value |
| --- | --- | --- | --- | --- | --- |
| NC vs. IMQ | -14.68 | -16.65 to -12.71 | Yes | **** | <0.0001 |
| NC vs. G | -11.19 | -13.16 to -9.222 | Yes | **** | <0.0001 |
| NC vs. NS | -9.767 | -11.74 to -7.797 | Yes | **** | <0.0001 |
| NC vs. NM | -4.432 | -6.402 to -2.462 | Yes | **** | <0.0001 |
| NC vs. NL | -2.140 | -4.110 to -0.1702 | Yes | * | 0.0271 |
| IMQ vs. G | 3.488 | 1.518 to 5.458 | Yes | *** | 0.0001 |
| IMQ vs. NS | 4.913 | 2.943 to 6.883 | Yes | **** | <0.0001 |
| IMQ vs. NM | 10.25 | 8.278 to 12.22 | Yes | **** | <0.0001 |
| IMQ vs. NL | 12.54 | 10.57 to 14.51 | Yes | **** | <0.0001 |
| G vs. NS | 1.425 | -0.5448 to 3.395 | No | ns | 0.2673 |
| G vs. NM | 6.760 | 4.790 to 8.730 | Yes | **** | <0.0001 |
| G vs. NL | 9.052 | 7.082 to 11.02 | Yes | **** | <0.0001 |
| NS vs. NM | 5.335 | 3.365 to 7.305 | Yes | **** | <0.0001 |
| NS vs. NL | 7.627 | 5.657 to 9.597 | Yes | **** | <0.0001 |
| NM vs. NL | 2.292 | 0.3218 to 4.262 | Yes | * | 0.0153 |

**Supplementary table 3: serum level of IL 23**

| Tukey's multiple comparisons test | Mean Diff. | 95.00% CI of diff. | Significant? | Summary | Adjusted P Value |
| --- | --- | --- | --- | --- | --- |
| NC vs. IMQ | -17.18 | -18.79 to -15.58 | Yes | **** | <0.0001 |
| NC vs. G | -10.24 | -11.85 to -8.638 | Yes | **** | <0.0001 |
| NC vs. NS | -8.852 | -10.46 to -7.248 | Yes | **** | <0.0001 |
| NC vs. NM | -4.143 | -5.747 to -2.540 | Yes | **** | <0.0001 |
| NC vs. NL | -1.727 | -3.330 to -0.1229 | Yes | * | 0.0291 |
| IMQ vs. G | 6.940 | 5.336 to 8.544 | Yes | **** | <0.0001 |
| IMQ vs. NS | 8.330 | 6.726 to 9.934 | Yes | **** | <0.0001 |
| IMQ vs. NM | 13.04 | 11.43 to 14.64 | Yes | **** | <0.0001 |
| IMQ vs. NL | 15.46 | 13.85 to 17.06 | Yes | **** | <0.0001 |
| G vs. NS | 1.390 | -0.2137 to 2.994 | No | ns | 0.1195 |
| G vs. NM | 6.098 | 4.495 to 7.702 | Yes | **** | <0.0001 |
| G vs. NL | 8.515 | 6.911 to 10.12 | Yes | **** | <0.0001 |
| NS vs. NM | 4.708 | 3.105 to 6.312 | Yes | **** | <0.0001 |
| NS vs. NL | 7.125 | 5.521 to 8.729 | Yes | **** | <0.0001 |
| NM vs. NL | 2.417 | 0.8129 to 4.020 | Yes | *** | 0.0010 |

**Supplementary table 4: TLR7**

| Tukey's multiple comparisons test | Mean Diff. | 95.00% CI of diff. | Significant? | Summary | Adjusted P Value |
| --- | --- | --- | --- | --- | --- |
| NC vs. IMQ | -4.645 | -5.101 to -4.189 | Yes | **** | <0.0001 |
| NC vs. G | -2.645 | -3.101 to -2.189 | Yes | **** | <0.0001 |
| NC vs. NS | -2.820 | -3.276 to -2.364 | Yes | **** | <0.0001 |
| NC vs. NM | -1.495 | -1.951 to -1.039 | Yes | **** | <0.0001 |
| NC vs. NL | -0.8750 | -1.331 to -0.4193 | Yes | *** | 0.0001 |
| IMQ vs. G | 2.000 | 1.544 to 2.456 | Yes | **** | <0.0001 |
| IMQ vs. NS | 1.825 | 1.369 to 2.281 | Yes | **** | <0.0001 |
| IMQ vs. NM | 3.150 | 2.694 to 3.606 | Yes | **** | <0.0001 |
| IMQ vs. NL | 3.770 | 3.314 to 4.226 | Yes | **** | <0.0001 |
| G vs. NS | -0.1750 | -0.6307 to 0.2807 | No | ns | 0.8215 |
| G vs. NM | 1.150 | 0.6943 to 1.606 | Yes | **** | <0.0001 |
| G vs. NL | 1.770 | 1.314 to 2.226 | Yes | **** | <0.0001 |
| NS vs. NM | 1.325 | 0.8693 to 1.781 | Yes | **** | <0.0001 |
| NS vs. NL | 1.945 | 1.489 to 2.401 | Yes | **** | <0.0001 |
| NM vs. NL | 0.6200 | 0.1643 to 1.076 | Yes | ** | 0.0047 |

**Supplementary table 5: TLR8**

| Tukey's multiple comparisons test | Mean Diff. | 95.00% CI of diff. | Significant? | Summary | Adjusted P Value |
| --- | --- | --- | --- | --- | --- |
| NC vs. IMQ | -5.585 | -6.025 to -5.145 | Yes | **** | <0.0001 |
| NC vs. G | -2.940 | -3.380 to -2.500 | Yes | **** | <0.0001 |
| NC vs. NS | -2.785 | -3.225 to -2.345 | Yes | **** | <0.0001 |
| NC vs. NM | -1.235 | -1.675 to -0.7951 | Yes | **** | <0.0001 |
| NC vs. NL | -0.4600 | -0.8999 to -0.02012 | Yes | * | 0.0374 |
| IMQ vs. G | 2.645 | 2.205 to 3.085 | Yes | **** | <0.0001 |
| IMQ vs. NS | 2.800 | 2.360 to 3.240 | Yes | **** | <0.0001 |
| IMQ vs. NM | 4.350 | 3.910 to 4.790 | Yes | **** | <0.0001 |
| IMQ vs. NL | 5.125 | 4.685 to 5.565 | Yes | **** | <0.0001 |
| G vs. NS | 0.1550 | -0.2849 to 0.5949 | No | ns | 0.8669 |
| G vs. NM | 1.705 | 1.265 to 2.145 | Yes | **** | <0.0001 |
| G vs. NL | 2.480 | 2.040 to 2.920 | Yes | **** | <0.0001 |
| NS vs. NM | 1.550 | 1.110 to 1.990 | Yes | **** | <0.0001 |
| NS vs. NL | 2.325 | 1.885 to 2.765 | Yes | **** | <0.0001 |
| NM vs. NL | 0.7750 | 0.3351 to 1.215 | Yes | *** | 0.0003 |

**Supplementary table 6: epidermal sickness**

| Tukey's multiple comparisons test | Mean Diff. | 95.00% CI of diff. | Significant? | Summary | Adjusted P Value |
| --- | --- | --- | --- | --- | --- |
| NC vs. IMQ | -322.9 | -383.8 to -261.9 | Yes | **** | <0.0001 |
| NC vs. G | -207.9 | -268.9 to -147.0 | Yes | **** | <0.0001 |
| NC vs. NS | -192.9 | -253.9 to -132.0 | Yes | **** | <0.0001 |
| NC vs. NM | -130.2 | -191.1 to -69.18 | Yes | **** | <0.0001 |
| NC vs. NL | -61.03 | -122.0 to -0.05167 | Yes | * | 0.0497 |
| IMQ vs. G | 114.9 | 53.94 to 175.9 | Yes | **** | <0.0001 |
| IMQ vs. NS | 129.9 | 68.95 to 190.9 | Yes | **** | <0.0001 |
| IMQ vs. NM | 192.7 | 131.7 to 253.7 | Yes | **** | <0.0001 |
| IMQ vs. NL | 261.8 | 200.9 to 322.8 | Yes | **** | <0.0001 |
| G vs. NS | 15.01 | -45.96 to 75.99 | No | ns | 0.9714 |
| G vs. NM | 77.79 | 16.82 to 138.8 | Yes | ** | 0.0071 |
| G vs. NL | 146.9 | 85.94 to 207.9 | Yes | **** | <0.0001 |
| NS vs. NM | 62.78 | 1.801 to 123.8 | Yes | * | 0.0410 |
| NS vs. NL | 131.9 | 70.93 to 192.9 | Yes | **** | <0.0001 |
| NM vs. NL | 69.13 | 8.153 to 130.1 | Yes | * | 0.0199 |

**Supplementary table 7: serum level of Foxp3**

| Tukey's multiple comparisons test | Mean Diff. | 95.00% CI of diff. | Significant? | Summary | Adjusted P Value |
| --- | --- | --- | --- | --- | --- |
| NC vs. IMQ | -2.768 | -4.510 to -1.026 | Yes | *** | 0.0007 |
| NC vs. G | -6.400 | -8.142 to -4.658 | Yes | **** | <0.0001 |
| NC vs. NS | -6.400 | -8.142 to -4.658 | Yes | **** | <0.0001 |
| NC vs. NM | -9.000 | -10.74 to -7.258 | Yes | **** | <0.0001 |
| NC vs. NL | -11.71 | -13.45 to -9.966 | Yes | **** | <0.0001 |
| IMQ vs. G | -3.632 | -5.374 to -1.890 | Yes | **** | <0.0001 |
| IMQ vs. NS | -3.632 | -5.374 to -1.890 | Yes | **** | <0.0001 |
| IMQ vs. NM | -6.232 | -7.974 to -4.490 | Yes | **** | <0.0001 |
| IMQ vs. NL | -8.940 | -10.68 to -7.198 | Yes | **** | <0.0001 |
| G vs. NS | 0.000 | -1.742 to 1.742 | No | ns | >0.9999 |
| G vs. NM | -2.600 | -4.342 to -0.8584 | Yes | ** | 0.0014 |
| G vs. NL | -5.308 | -7.050 to -3.566 | Yes | **** | <0.0001 |
| NS vs. NM | -2.600 | -4.342 to -0.8584 | Yes | ** | 0.0014 |
| NS vs. NL | -5.308 | -7.050 to -3.566 | Yes | **** | <0.0001 |
| NM vs. NL | -2.708 | -4.450 to -0.9664 | Yes | *** | 0.0009 |

**Supplementary table 8: RORg**

| Tukey's multiple comparisons test | Mean Diff. | 95.00% CI of diff. | Significant? | Summary | Adjusted P Value |
| --- | --- | --- | --- | --- | --- |
| NC vs. IMQ | -12.60 | -14.20 to -11.00 | Yes | **** | <0.0001 |
| NC vs. G | -9.800 | -11.40 to -8.203 | Yes | **** | <0.0001 |
| NC vs. NS | -9.600 | -11.20 to -8.003 | Yes | **** | <0.0001 |
| NC vs. NM | -6.800 | -8.397 to -5.203 | Yes | **** | <0.0001 |
| NC vs. NL | -3.000 | -4.597 to -1.403 | Yes | **** | <0.0001 |
| IMQ vs. G | 2.800 | 1.203 to 4.397 | Yes | *** | 0.0002 |
| IMQ vs. NS | 3.000 | 1.403 to 4.597 | Yes | **** | <0.0001 |
| IMQ vs. NM | 5.800 | 4.203 to 7.397 | Yes | **** | <0.0001 |
| IMQ vs. NL | 9.600 | 8.003 to 11.20 | Yes | **** | <0.0001 |
| G vs. NS | 0.2000 | -1.397 to 1.797 | No | ns | 0.9987 |
| G vs. NM | 3.000 | 1.403 to 4.597 | Yes | **** | <0.0001 |
| G vs. NL | 6.800 | 5.203 to 8.397 | Yes | **** | <0.0001 |
| NS vs. NM | 2.800 | 1.203 to 4.397 | Yes | *** | 0.0002 |
| NS vs. NL | 6.600 | 5.003 to 8.197 | Yes | **** | <0.0001 |
| NM vs. NL | 3.800 | 2.203 to 5.397 | Yes | **** | <0.0001 |

**Supplementary table 9: PCNA**

| Tukey's multiple comparisons test | Mean Diff. | 95.00% CI of diff. | Significant? | Summary | Adjusted P Value |
| --- | --- | --- | --- | --- | --- |
| NC vs. IMQ | -6.730 | -7.317 to -6.143 | Yes | **** | <0.0001 |
| NC vs. G | -3.010 | -3.597 to -2.423 | Yes | **** | <0.0001 |
| NC vs. NS | -2.940 | -3.527 to -2.353 | Yes | **** | <0.0001 |
| NC vs. NM | -1.580 | -2.167 to -0.9934 | Yes | **** | <0.0001 |
| NC vs. NL | -0.9800 | -1.567 to -0.3934 | Yes | *** | 0.0006 |
| IMQ vs. G | 3.720 | 3.133 to 4.307 | Yes | **** | <0.0001 |
| IMQ vs. NS | 3.790 | 3.203 to 4.377 | Yes | **** | <0.0001 |
| IMQ vs. NM | 5.150 | 4.563 to 5.737 | Yes | **** | <0.0001 |
| IMQ vs. NL | 5.750 | 5.163 to 6.337 | Yes | **** | <0.0001 |
| G vs. NS | 0.07000 | -0.5166 to 0.6566 | No | ns | 0.9988 |
| G vs. NM | 1.430 | 0.8434 to 2.017 | Yes | **** | <0.0001 |
| G vs. NL | 2.030 | 1.443 to 2.617 | Yes | **** | <0.0001 |
| NS vs. NM | 1.360 | 0.7734 to 1.947 | Yes | **** | <0.0001 |
| NS vs. NL | 1.960 | 1.373 to 2.547 | Yes | **** | <0.0001 |
| NM vs. NL | 0.6000 | 0.01343 to 1.187 | Yes | * | 0.0433 |

**Supplementary table 10: KI-67**

| Tukey's multiple comparisons test | Mean Diff. | 95.00% CI of diff. | Significant? | Summary | Adjusted P Value |
| --- | --- | --- | --- | --- | --- |
| NC vs. IMQ | -5.585 | -6.006 to -5.164 | Yes | **** | <0.0001 |
| NC vs. G | -2.685 | -3.106 to -2.264 | Yes | **** | <0.0001 |
| NC vs. NS | -2.335 | -2.756 to -1.914 | Yes | **** | <0.0001 |
| NC vs. NM | -1.585 | -2.006 to -1.164 | Yes | **** | <0.0001 |
| NC vs. NL | -0.7600 | -1.181 to -0.3387 | Yes | *** | 0.0002 |
| IMQ vs. G | 2.900 | 2.479 to 3.321 | Yes | **** | <0.0001 |
| IMQ vs. NS | 3.250 | 2.829 to 3.671 | Yes | **** | <0.0001 |
| IMQ vs. NM | 4.000 | 3.579 to 4.421 | Yes | **** | <0.0001 |
| IMQ vs. NL | 4.825 | 4.404 to 5.246 | Yes | **** | <0.0001 |
| G vs. NS | 0.3500 | -0.07128 to 0.7713 | No | ns | 0.1378 |
| G vs. NM | 1.100 | 0.6787 to 1.521 | Yes | **** | <0.0001 |
| G vs. NL | 1.925 | 1.504 to 2.346 | Yes | **** | <0.0001 |
| NS vs. NM | 0.7500 | 0.3287 to 1.171 | Yes | *** | 0.0003 |
| NS vs. NL | 1.575 | 1.154 to 1.996 | Yes | **** | <0.0001 |
| NM vs. NL | 0.8250 | 0.4037 to 1.246 | Yes | **** | <0.0001 |

**Supplementary table 11: BCL2**

| Tukey's multiple comparisons test | Mean Diff. | 95.00% CI of diff. | Significant? | Summary | Adjusted P Value |
| --- | --- | --- | --- | --- | --- |
| NC vs. IMQ | -4.835 | -5.380 to -4.290 | Yes | **** | <0.0001 |
| NC vs. G | -2.635 | -3.180 to -2.090 | Yes | **** | <0.0001 |
| NC vs. NS | -2.585 | -3.130 to -2.040 | Yes | **** | <0.0001 |
| NC vs. NM | -1.735 | -2.280 to -1.190 | Yes | **** | <0.0001 |
| NC vs. NL | -0.8100 | -1.355 to -0.2653 | Yes | ** | 0.0020 |
| IMQ vs. G | 2.200 | 1.655 to 2.745 | Yes | **** | <0.0001 |
| IMQ vs. NS | 2.250 | 1.705 to 2.795 | Yes | **** | <0.0001 |
| IMQ vs. NM | 3.100 | 2.555 to 3.645 | Yes | **** | <0.0001 |
| IMQ vs. NL | 4.025 | 3.480 to 4.570 | Yes | **** | <0.0001 |
| G vs. NS | 0.05000 | -0.4947 to 0.5947 | No | ns | 0.9997 |
| G vs. NM | 0.9000 | 0.3553 to 1.445 | Yes | *** | 0.0007 |
| G vs. NL | 1.825 | 1.280 to 2.370 | Yes | **** | <0.0001 |
| NS vs. NM | 0.8500 | 0.3053 to 1.395 | Yes | ** | 0.0012 |
| NS vs. NL | 1.775 | 1.230 to 2.320 | Yes | **** | <0.0001 |
| NM vs. NL | 0.9250 | 0.3803 to 1.470 | Yes | *** | 0.0005 |

**Supplementary table 12: AKT**

| Tukey's multiple comparisons test | Mean Diff. | 95.00% CI of diff. | Significant? | Summary | Adjusted P Value |
| --- | --- | --- | --- | --- | --- |
| NC vs. IMQ | -3.585 | -3.942 to -3.228 | Yes | **** | <0.0001 |
| NC vs. G | -2.060 | -2.417 to -1.703 | Yes | **** | <0.0001 |
| NC vs. NS | -2.180 | -2.537 to -1.823 | Yes | **** | <0.0001 |
| NC vs. NM | -1.470 | -1.827 to -1.113 | Yes | **** | <0.0001 |
| NC vs. NL | -0.5225 | -0.8798 to -0.1652 | Yes | ** | 0.0023 |
| IMQ vs. G | 1.525 | 1.168 to 1.882 | Yes | **** | <0.0001 |
| IMQ vs. NS | 1.405 | 1.048 to 1.762 | Yes | **** | <0.0001 |
| IMQ vs. NM | 2.115 | 1.758 to 2.472 | Yes | **** | <0.0001 |
| IMQ vs. NL | 3.063 | 2.705 to 3.420 | Yes | **** | <0.0001 |
| G vs. NS | -0.1200 | -0.4773 to 0.2373 | No | ns | 0.8879 |
| G vs. NM | 0.5900 | 0.2327 to 0.9473 | Yes | *** | 0.0007 |
| G vs. NL | 1.538 | 1.180 to 1.895 | Yes | **** | <0.0001 |
| NS vs. NM | 0.7100 | 0.3527 to 1.067 | Yes | **** | <0.0001 |
| NS vs. NL | 1.658 | 1.300 to 2.015 | Yes | **** | <0.0001 |
| NM vs. NL | 0.9475 | 0.5902 to 1.305 | Yes | **** | <0.0001 |

**Supplementary table 13: TNF A IP3**

| Tukey's multiple comparisons test | Mean Diff. | 95.00% CI of diff. | Significant? | Summary | Adjusted P Value |  |
| --- | --- | --- | --- | --- | --- | --- |
| NC vs. IMQ | 0.7550 | 0.6843 to 0.8257 | Yes | **** | <0.0001 |  |
| NC vs. G | 0.4350 | 0.3643 to 0.5057 | Yes | **** | <0.0001 |  |
| NC vs. NS | 0.4700 | 0.3993 to 0.5407 | Yes | **** | <0.0001 |  |
| NC vs. NM | 0.3400 | 0.2693 to 0.4107 | Yes | **** | <0.0001 |  |
| NC vs. NL | 0.2000 | 0.1293 to 0.2707 | Yes | **** | <0.0001 |  |
| IMQ vs. G | -0.3200 | -0.3907 to -0.2493 | Yes | **** | <0.0001 |  |
| IMQ vs. NS | -0.2850 | -0.3557 to -0.2143 | Yes | **** | <0.0001 |  |
| IMQ vs. NM | -0.4150 | -0.4857 to -0.3443 | Yes | **** | <0.0001 |  |
| IMQ vs. NL | -0.5550 | -0.6257 to -0.4843 | Yes | **** | <0.0001 |  |
| G vs. NS | 0.03500 | -0.03567 to 0.1057 | No | ns | 0.6244 |  |
| G vs. NM | -0.09500 | -0.1657 to -0.02433 | Yes | ** | 0.0052 |  |
| G vs. NL | -0.2350 | -0.3057 to -0.1643 | Yes | **** | <0.0001 |  |
| NS vs. NM | -0.1300 | -0.2007 to -0.05933 | Yes | *** | 0.0002 |  |
| NS vs. NL | -0.2700 | -0.3407 to -0.1993 | Yes | **** | <0.0001 |  |
| NM vs. NL | -0.1400 | -0.2107 to -0.06933 | Yes | **** | <0.0001 |  |

**Supplementary table 14: NF-KB**

| Tukey's multiple comparisons test | Mean Diff. | 95.00% CI of diff. | Significant? | Summary | Adjusted P Value |
| --- | --- | --- | --- | --- | --- |
| NC vs. IMQ | -5.290 | -5.760 to -4.820 | Yes | **** | <0.0001 |
| NC vs. G | -1.690 | -2.160 to -1.220 | Yes | **** | <0.0001 |
| NC vs. NS | -1.590 | -2.060 to -1.120 | Yes | **** | <0.0001 |
| NC vs. NM | -1.115 | -1.585 to -0.6448 | Yes | **** | <0.0001 |
| NC vs. NL | -0.4900 | -0.9602 to -0.01984 | Yes | * | 0.0383 |
| IMQ vs. G | 3.600 | 3.130 to 4.070 | Yes | **** | <0.0001 |
| IMQ vs. NS | 3.700 | 3.230 to 4.170 | Yes | **** | <0.0001 |
| IMQ vs. NM | 4.175 | 3.705 to 4.645 | Yes | **** | <0.0001 |
| IMQ vs. NL | 4.800 | 4.330 to 5.270 | Yes | **** | <0.0001 |
| G vs. NS | 0.1000 | -0.3702 to 0.5702 | No | ns | 0.9825 |
| G vs. NM | 0.5750 | 0.1048 to 1.045 | Yes | * | 0.0117 |
| G vs. NL | 1.200 | 0.7298 to 1.670 | Yes | **** | <0.0001 |
| NS vs. NM | 0.4750 | 0.004843 to 0.9452 | Yes | * | 0.0469 |
| NS vs. NL | 1.100 | 0.6298 to 1.570 | Yes | **** | <0.0001 |
| NM vs. NL | 0.6250 | 0.1548 to 1.095 | Yes | ** | 0.0057 |

**Supplementary table 15: miRNA 31**

| Tukey's multiple comparisons test | Mean Diff. | 95.00% CI of diff. | Significant? | Summary | Adjusted P Value |
| --- | --- | --- | --- | --- | --- |
| NC vs. IMQ | -7.415 | -8.019 to -6.811 | Yes | **** | <0.0001 |
| NC vs. G | -3.580 | -4.184 to -2.976 | Yes | **** | <0.0001 |
| NC vs. NS | -3.365 | -3.969 to -2.761 | Yes | **** | <0.0001 |
| NC vs. NM | -2.265 | -2.869 to -1.661 | Yes | **** | <0.0001 |
| NC vs. NL | -0.9800 | -1.584 to -0.3761 | Yes | *** | 0.0008 |
| IMQ vs. G | 3.835 | 3.231 to 4.439 | Yes | **** | <0.0001 |
| IMQ vs. NS | 4.050 | 3.446 to 4.654 | Yes | **** | <0.0001 |
| IMQ vs. NM | 5.150 | 4.546 to 5.754 | Yes | **** | <0.0001 |
| IMQ vs. NL | 6.435 | 5.831 to 7.039 | Yes | **** | <0.0001 |
| G vs. NS | 0.2150 | -0.3889 to 0.8189 | No | ns | 0.8620 |
| G vs. NM | 1.315 | 0.7111 to 1.919 | Yes | **** | <0.0001 |
| G vs. NL | 2.600 | 1.996 to 3.204 | Yes | **** | <0.0001 |
| NS vs. NM | 1.100 | 0.4961 to 1.704 | Yes | *** | 0.0002 |
| NS vs. NL | 2.385 | 1.781 to 2.989 | Yes | **** | <0.0001 |
| NM vs. NL | 1.285 | 0.6811 to 1.889 | Yes | **** | <0.0001 |

**Supplementary table 16: P70S6k**

| Tukey's multiple comparisons test | Mean Diff. | 95.00% CI of diff. | Significant? | Summary | Adjusted P Value |
| --- | --- | --- | --- | --- | --- |
| NC vs. IMQ | -6.620 | -7.278 to -5.962 | Yes | **** | <0.0001 |
| NC vs. G | -3.490 | -4.148 to -2.832 | Yes | **** | <0.0001 |
| NC vs. NS | -3.615 | -4.273 to -2.957 | Yes | **** | <0.0001 |
| NC vs. NM | -2.715 | -3.373 to -2.057 | Yes | **** | <0.0001 |
| NC vs. NL | -1.990 | -2.648 to -1.332 | Yes | **** | <0.0001 |
| IMQ vs. G | 3.130 | 2.472 to 3.788 | Yes | **** | <0.0001 |
| IMQ vs. NS | 3.005 | 2.347 to 3.663 | Yes | **** | <0.0001 |
| IMQ vs. NM | 3.905 | 3.247 to 4.563 | Yes | **** | <0.0001 |
| IMQ vs. NL | 4.630 | 3.972 to 5.288 | Yes | **** | <0.0001 |
| G vs. NS | -0.1250 | -0.7834 to 0.5334 | No | ns | 0.9894 |
| G vs. NM | 0.7750 | 0.1166 to 1.433 | Yes | * | 0.0159 |
| G vs. NL | 1.500 | 0.8416 to 2.158 | Yes | **** | <0.0001 |
| NS vs. NM | 0.9000 | 0.2416 to 1.558 | Yes | ** | 0.0045 |
| NS vs. NL | 1.625 | 0.9666 to 2.283 | Yes | **** | <0.0001 |
| NM vs. NL | 0.7250 | 0.06658 to 1.383 | Yes | * | 0.0262 |
